# Novel strategies for cancer immunotherapy: counter-immunoediting therapy

**DOI:** 10.1186/s13045-023-01430-8

**Published:** 2023-04-13

**Authors:** Shaochuan Liu, Qian Sun, Xiubao Ren

**Affiliations:** 1 Department of Immunology, Tianjin Medical University Cancer Institute and Hospital, 300060 Tianjin, China; 2 Tianjin Medical University Cancer Institute & Hospital, National Clinical Research Center for Cancer, 300060 Tianjin, China; 3 Key Laboratory of Cancer Immunology and Biotherapy, 300060 Tianjin, China; 4 Key Laboratory of Cancer Prevention and Therapy, 300060 Tianjin, China; 5 Tianjin’s Clinical Research Center for Cancer, 300060 Tianjin, China; 6 Department of Biotherapy, Tianjin Medical University Cancer Institute and Hospital, 300060 Tianjin, China

## Abstract

The advent of immunotherapy has made an indelible mark on the field of cancer therapy, especially the application of immune checkpoint inhibitors in clinical practice. Although immunotherapy has proven its efficacy and safety in some tumors, many patients still have innate or acquired resistance to immunotherapy. The emergence of this phenomenon is closely related to the highly heterogeneous immune microenvironment formed by tumor cells after undergoing cancer immunoediting. The process of cancer immunoediting refers to the cooperative interaction between tumor cells and the immune system that involves three phases: elimination, equilibrium, and escape. During these phases, conflicting interactions between the immune system and tumor cells result in the formation of a complex immune microenvironment, which contributes to the acquisition of different levels of immunotherapy resistance in tumor cells. In this review, we summarize the characteristics of different phases of cancer immunoediting and the corresponding therapeutic tools, and we propose normalized therapeutic strategies based on immunophenotyping. The process of cancer immunoediting is retrograded through targeted interventions in different phases of cancer immunoediting, making immunotherapy in the context of precision therapy the most promising therapy to cure cancer.

## Background

**Fig. 1:**
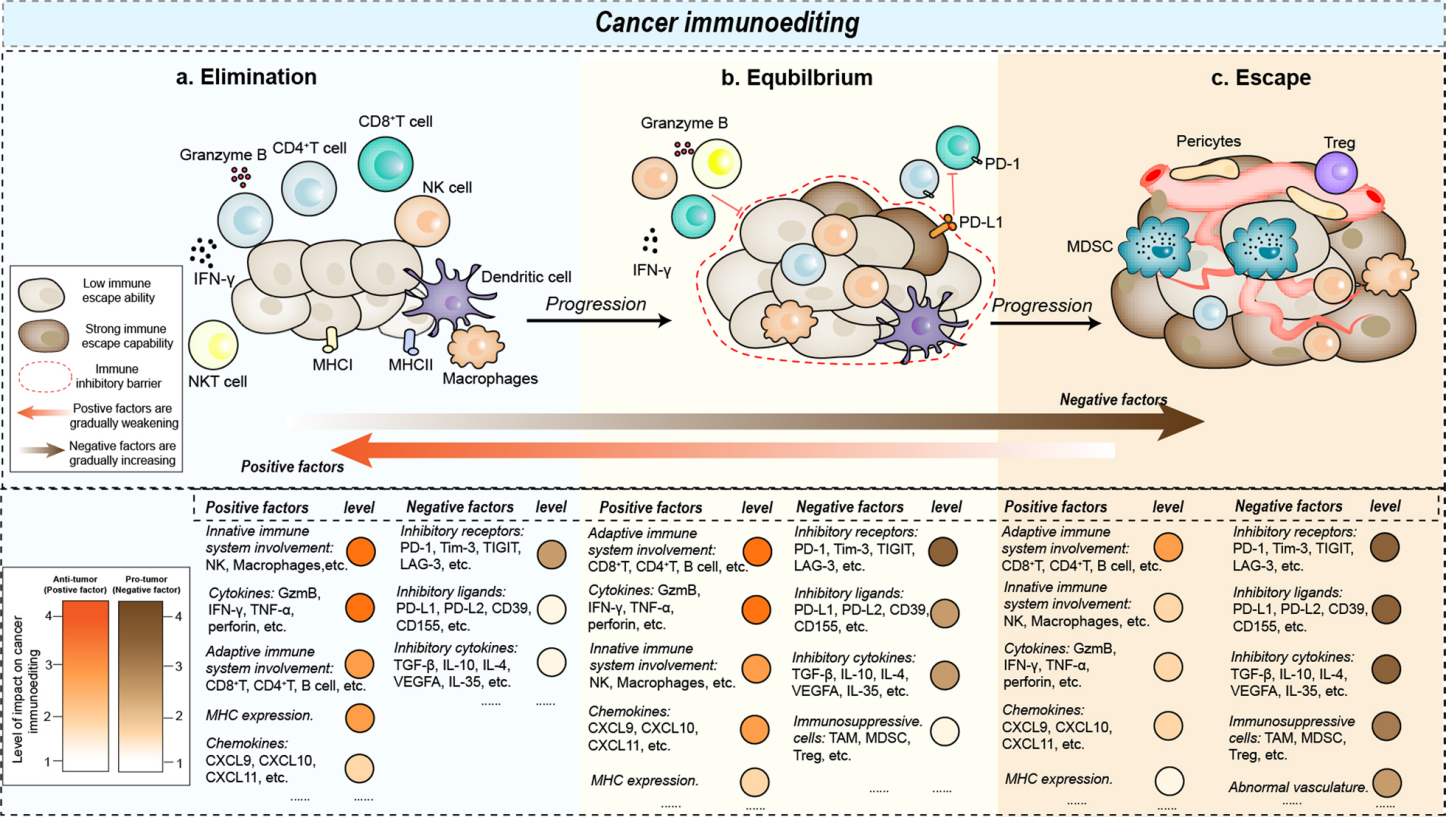
The three phases of cancer immunoediting: elimination, equilibrium, and escape. **a** During the elimination phase, the innate and adaptive immune systems synergize to identify and eliminate malignant or transformed tumor cells before clinical detection. **b** During the equilibrium phase, a relative balance is established between the tumor cells and the immune system, with the immune system unable to completely eliminate the tumor cells and the tumor cells unable to evade immune surveillance. **c** During the escape phase, tumor cell growth and proliferation are no longer restricted by the immune system. The accumulation of rapidly proliferating tumor cells in combination with other stromal cells creates a more complex immunosuppressive microenvironment, thus further damaging the balance between tumor cells and the immune system. During the proceeding of cancer immunoediting, the ability of the immune system to monitor, recognize, and kill tumor cells is crucial in halting its progression. Factors that enhance this ability are positive, while those that enable tumor cells to evade immune recognition and killing are negative. The impact of these factors in the process of cancer immunoediting has been quantified and classified as strong expression (score 4), moderate intensity expression (score 3), weak expression (score 2), and pianissimo expression (score 1). TAMs, tumor-associated macrophages; MDSCs, nyeloid-derived suppressor cells; MHC, major histocompatibility complex; GzmB: Granzyme B

The concept of cancer immunoediting was first introduced by Schreiber in 2002; after years of research and revisions, the present theory of cancer immunoediting was finally developed [1]. This theory suggests that the immune system can paradoxically inhibit or promote the formation of tumor tissue, and this process is regulated in a complex mechanism comprising three main phases: “elimination,” “equilibrium,” and “escape” (Fig. 1). During the elimination phase, the innate and adaptive immune systems synergize to identify malignant or transformed tumor cells and eliminate them before they can be clinically detected. If some of the tumor cells survive the elimination phase due to low immunogenicity or other reasons, they enter the equilibrium phase, in which tumor cell proliferation is arrested but the tumor cells are present. During this phase, the interaction between the adaptive immune system and tumor cells is maintained in a stable equilibrium for a long period of time, which can last for years or decades. When tumor cells undergo genetic mutations or are stimulated by other factors, they pass from the equilibrium phase to the escape phase, in which their growth can no longer be controlled. Furthermore, tumor cells in the escape phase express a variety of immunosuppressive ligands to inhibit the function of effector T cells and escape the attack of immune cells. The theory of cancer immunoediting has become increasingly sophisticated, but in the process of cancer immunoediting, all three phases are not necessarily experienced. Indeed, some tumors may not enter the equilibrium phase and move directly to the escape phase, while others may never enter the escape phase and remain in equilibrium.

Despite advances in cancer detection, tumors in the elimination and equilibrium phases are generally undetectable. As a result, most tumors are already in the escape phase by the time they are clinically diagnosed [2]. At this stage, the intricate immune microenvironment, formed by conflicting interactions between tumor cells and the immune system, hinders the efficacy of existing therapies, as in the cases of pancreatic and small cell lung cancers [3, 4].Therefore, the development of effective interventions for tumors in the escape phase has become an urgent clinical need. Although novel therapeutic approaches, including CAR-T and anti-PD-1 therapy, have been developed to address malignant tumor progression, the treatment paradigm still follows the traditional model of single-target and single-drug therapy, often failing to fully consider the cancer immunoediting process. This shortcoming is one of the reasons for the unsatisfactory efficacy of cancer therapy. The cancer immunoediting process is often recurrent and insidious, and the immune characteristics of different cancer immunoediting stages often differ, particularly those of tumors in the escape phase, which are more complex. Therefore, a systematic and precise treatment strategy is urgently needed to effectively identify the current cancer immunoediting phase and immune characteristics of tumors, and provide appropriate and effective interventions to counter or even reverse the process of cancer immunoediting.

Based on this, we have summarized the characteristics and clinical features of the several cancer immunoediting phases and proposed a new therapeutic concept based on the theory of cancer immunoediting, i.e., a set of precise clinical therapeutic interventions tailored for different cancer immunoediting phases to counter or even reverse the process of cancer immunoediting. As a result, tumors in the escape phase can be reverted into the equilibrium or even elimination phase and can still receive precise therapeutic interventions during these phases, thus maximizing the patient's ability to achieve complete remission (CR) and maintain a long-term cure. We have named this therapeutic concept "counter-immunoediting therapy".

### Characteristics and counter-immunoediting strategies in the escape phase

Of the cancer immunoediting phases, the escape phase is the one that is most readily observable within the clinical setting. Several reviews have discussed the process of cancer immunoediting in tumor cells during the escape phase, as well as dynamic interactions with immune cells [5, 6]. During the escape phase, the balance between tumor cells and the immune system is damaged, the growth rate of tumor cells is uncontrolled, and large numbers of abnormally growing tumor cells and stromal cells develop an immunosuppressive tumor microenvironment (TME). For example, in response to hypoxia, large amounts of vascular endothelial growth factor (VEGF) A are released that bind to VEGF receptor 2 on endothelial cells, thus promoting neovascularization and leading to the rapid development of cancer. A large number of distorted and deformed endothelial cells constitute abnormal vasculature, making it much more difficult for immune cells to infiltrate into tumor tissues [7]. In addition, VEGFA inhibits the maturation of dendritic cells (DCs) [8], promotes infiltration of regulatory T cells (Treg cells) [9] and myeloid-derived suppressor cells (MDSCs) [10], thus accelerating the formation of an immunosuppressive environment. Tumors themselves also express multiple immunosuppressive ligands [11, 12] and downregulate major histocompatibility complex (MHC) class I molecules expression to evade recognition and killing by immune cells [13]. The presence of multiple immunosuppressive factors exacerbates the imbalance between tumor cells and the immune system, ultimately leading to uncontrolled tumor growth and endangering patient survival.

The aim of treatment at this phase is to inhibit the continuous growth of tumor tissues, restore the balance between the immune system and tumor cells in tumor sites, and even achieve the complete elimination of tumor cells. The current treatment and paradigm of tumor therapy, especially for mid- to advanced-stage tumors, are still determined based on standards such as cancer type, lymph node metastasis, and clinical stage, and they rarely consider the infiltration of immune cells into the tumor tissue. Based on the theory of cancer immunoediting, the infiltration of immune cells into tumor tissues should not be excluded from the establishment of tumor treatment protocols, and the function of the immune cells themselves should be further analyzed so that the immune system in tumor tissues can be comprehensively evaluated and targeted in order to formulate precise counter-immunoediting strategies [5, 14–16].

### Identifying the tumor immunophenotype in the escape phase

As technology continues to advance, researchers and physicians have shifted from relying solely on traditional immunohistochemical evaluation of tumor tissue to a more comprehensive approach. A combination of multiplex immunohistochemistry, single-cell sequencing, and spatial transcriptome technologies to assess tumor status and analyze immune infiltration in tumor tissues can be used to develop corresponding treatment plans. Halima et al. previously summarized cancer immunotherapy biomarkers discovered using high-throughput sequencing technology and highlighted the importance of utilizing such biomarkers for individualized therapy [17]. However, there is no uniform standard for immunophenotyping in tumor tissues and its application to clinical practice.

In an early exploration of the TME, researchers found a strong link between tumor progression and the immune system, which they used to develop a new immune evaluation system called the immune score [18–20]. The TME can be quantified and classified using immune scoring of CD3^+^ and CD8^+^ T lymphocytes infiltrating the tumor center or tumor margin. These can be broadly classified into two categories: those with T-cell infiltration and inflammation are categorized as hot tumors, and those with T-cell deficiency or exclusion are categorized as cold tumors [21, 22]. Malka et al. summarized several immune scoring methods commonly used for colorectal cancer, and one of the standard methods to assess immune cell infiltration was proposed by Professor Galon, named Immunoscore®, based on a multicenter clinical trial [23, 24]. Immunoscore® defines hot and cold tumors with a higher prognostic value than pathological TNM staging, lymphovascular invasion, tumor differentiation, and microsatellite instability (MSI) status [24]. With additional research, Galon et al. further classified the tumor immune microenvironment (TIME) into four types, the first being hot tumors, in which the tumor tissue is infiltrated with a large number of T cells and cytotoxic T lymphocytes (CTL), with elevated expression of various immunosuppressive receptors on immune cells, such as programmed cell death protein 1 (PD-1), T-cell immunoglobulin (Ig) domain and mucin domain 3 (Tim-3), CTL-associated protein 4 (CTLA-4), and lymphocyte activation gene 3 (LAG-3). The second type is immunosuppressive tumors, which have a moderate infiltration of T cells but a large number of immunosuppressive factors (transforming growth factor β [TGF-β], VEGFA, and IL-10), and a large number of immunosuppressive cells (MDSCs, Treg cells, etc.) are present in the tumor tissue. The third type is immune-excluded tumors, where there is little or no T-cell infiltration in the tumor tissue, and most of the T cells are accumulated at the edge of the tumor tissue. The fourth type is cold tumors with minimal T cells within the tumor or at the tumor margin (low immune score), low tumor mutation burden (TMB), poor antigen presentation, and tumor cells that are resistant to T-cell killing [25]. In the inflammatory immune microenvironment, increased interferon (IFN) γ release due to increased CTL infiltration induces programmed cell death-ligand 1 (PD-L1) expression on tumor and stromal cells and is therefore sensitive to immune checkpoint inhibitors (ICIs) therapy [26]. It has also been suggested that the inflammatory environment itself can induce immunogenic death of tumor cells, such as scorching and apoptosis, thereby contributing to the sensitivity of tumor tissue to ICIs [27]. If ICI therapy is chosen without considering immunophenotyping, not only is there a possibility that the therapeutic effect will be greatly reduced, but the process of tumor development will also be accelerated [28]. Therefore, immunophenotyping not only helps researchers to have a good understanding of the immune landscape within the TME, but it also helps them develop personalized and precise medicine protocols for different immunophenotypes.

**Fig. 2:**
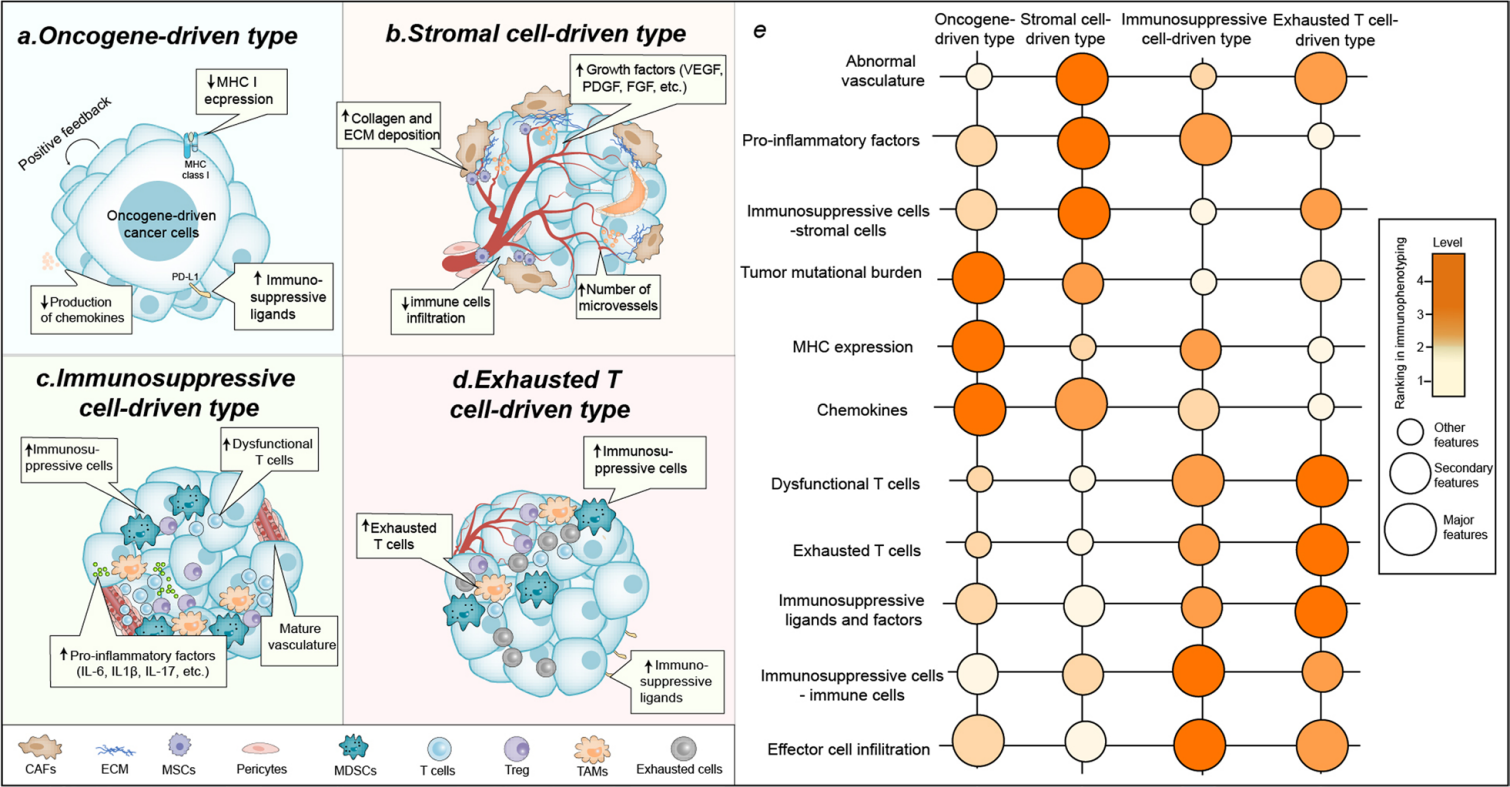
Four immunophenotypes of tumor microenvironment based on the driving factors. **a** Oncogene-driven type, **b** Stromal cell-driven type, **c** Immunsuppressive cell-driven type, and **d** Exhausted T cell-driven type. **e** We summarized the characteristics of each tumor type and ranked the importance of these characteristics in the different types. *TAMs* Tumor-associated macrophages, *CAFs* Tumor-associated fibroblasts, *MDSCs* Myeloid-derived suppressor cells, *MSCs* Mesenchymal Stem Cell, *ECM* Extracellular matrix, *MHC* Major histocompatibility complex

Therefore, based on the theory of cancer immunoediting and the 14 hallmarks of cancer proposed by Professor Hanahan [29], we interrelated the different cells affecting the TME with these 14 characteristics to reclassify tumor immunophenotypes into four types (Fig. 2).

The four types are the oncogene-driven type, exhausted T cell-driven type, immunosuppressive cell-driven type, and stromal cell-driven type.

- 1. Oncogene-driven type: In oncogene-driven immune microenvironments, tumor cells are endowed with rapid proliferation, resistance to apoptosis, increased expression of inhibitory checkpoints, and the induction of angiogenesis in the presence of driver genes; this is more common in oncogene-driven tumor tissues such as epidermal growth factor receptor (EGFR)-driven tumors, MYC-driven tumors, and Kirsten rat sarcoma (KRAS)-driven tumors [30–32]. With these characteristics, a TIME dominated by low TMB, MHC expression, and chemokine levels is formed [33–35]. This type of TIME belongs to the cold tumor type, which is poorly treated with ICI therapy alone due to the lack of sufficient infiltration of effector cells.
- 2. Stromal cell-driven type (more common in hepatocellular carcinoma, pancreatic cancer, and renal clear cell carcinoma): In stromal cell-driven tumors, a large number of stromal cells, including vascular endothelial cells (VECs), fibroblasts, pericytes, and mesenchymal cells, are present, and these stromal cells, together with immature neovascularization lacking pericyte coverage, form a hypoxic and metabolically abnormal TIME [36]. This type of TIME, which is also a cold tumor type, is resistant to ICI therapy due to the abnormal vascular network, which leads to difficulties in effector cell infiltration.
- 3. Immunosuppressive cell-driven type (more common in pancreatic cancer, prostate cancer, and breast cancer ): Immunosuppressive cells (tumor-associated macrophages [TAMs], MDSCs, regulatory B [Breg], Treg cells, etc.) mainly play the role of supporting tumor cell growth and immune escape, forming a TIME dominated by immunosuppressive cells, with low effector cell infiltration and dysfunction of effector cells. This type of TIME belongs to the category of hot tumors, which are better treated with ICI therapy.
- 4. Exhausted T cell-driven type (more common in tumors that are resistant to immunotherapy, such as glioblastoma [37]): In this type of TIME, effector cells, i.e., T cells, natural killer (NK) cells, B cells, etc., gradually lose their effector function and finally transform into exhausted cells in response to chronic inflammation and long-term tumor antigen stimulation [38]. In the presence of these exhausted T cells, an immune microenvironment with high expression of immunosuppressive factors and increased dysfunctional effector cells is formed, which is also a hot tumor type. These exhausted cells, especially terminally exhausted T cells, are resistant to ICI therapy [39].

Reclassifying the TME will help researchers understand the differences between the different TIMEs and develop tailored treatment regimens with greater potential for improving patient outcomes.

### Normalization strategies for stromal cells

**Fig. 3:**
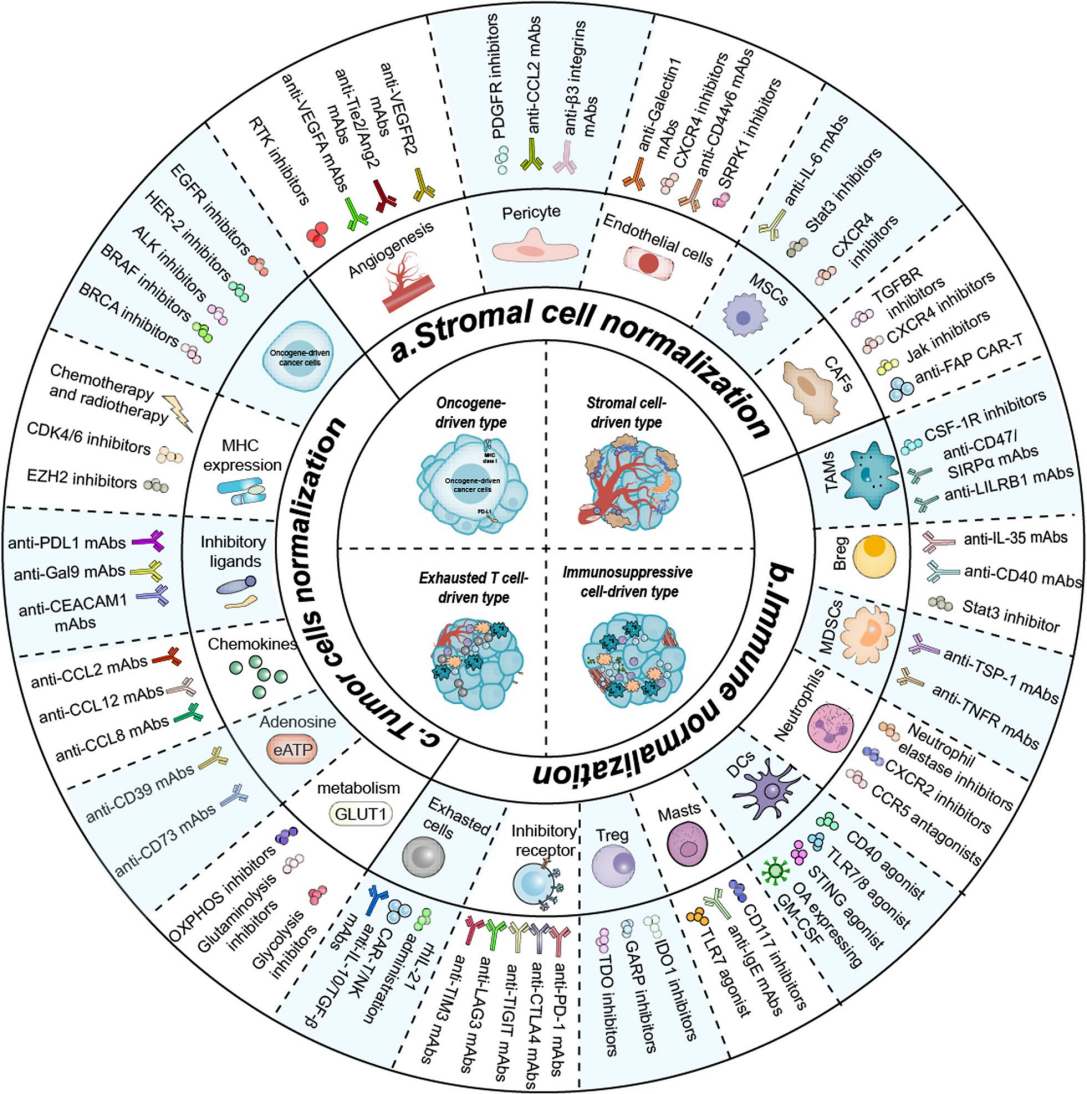
Pattern diagram of normalized treatment strategies targeting four tumor immunophenotypes. **a** Normalization of stromal cells, a therapeutic strategy to counter the proceeding of cancer immunoediting by targeting multiple stromal cells in the TME. **b** Normalization of immunity, a therapeutic strategy to counter the proceeding of cancer immunoediting by targeting multiple immunosuppressive factors in the TME. **c** Normalization of tumor cells, a therapeutic strategy to counter the proceeding of cancer immunoediting by targeting tumor cells and related factors in the TME. *TAMs* Tumor-associated macrophages, *CAFs* Tumor-associated fibroblasts, *MDSCs* Myeloid-derived suppressor cells, *MSCs* Mesenchymal Stem Cell, *ECM* Extracellular matrix, *MHC* Major histocompatibility complex, *DCs* Dendritic cells, *RTKs* Receptor tyrosine kinase inhibitors, *OA* Oncolytic adenovirus

In the TME, a large number of stromal cells collaborate with tumor cells to form an abnormal immune microenvironment, including, among others, cancer-associated fibroblasts (CAFs), VECs, and pericytes. These stromal cells can participate in the regulation of the immune system by expressing a variety of immunosuppressive factors and ligands to help tumor cells evade immune cell attack. The performance of stromal normalization strategies will greatly improve the tumor immunosuppressive microenvironment and enhance the infiltration of effector cells. This stromal normalization strategy is applicable to both oncogene-driven and stromal cell-driven tumors, and it can be used to target the corresponding cells as described in Fig. 3 to counter the process of cancer immunoediting.

#### Targeting vascular endothelial cells and neovascularization

The hypoxia and low pH of tumor tissues lead to pathological folding and distortion of the blood vessels without branching and easy leakage, which obstructs the infiltration of immune cells from the circulatory system into the tumor tissue and results in the formation of an immunosuppressive microenvironment [40]. In addition, VECs, stimulated by vascular growth factors, also express immunosuppressive molecules and do not respond to immune cells, forming an immunosuppressive barrier [41, 42]. The concept of vascular normalization was first put forward by Professor Jain in 2001, i.e., the application of anti-angiogenic therapy to target abnormal tumor vasculature to restore the normal physiological structure of tumor vessels and their ability to supply oxygen and drug transportation [43, 44].

Early anti-angiogenic drugs, mainly single-target drugs against VEGF/VEFGR, are ineffective, and many patients exhibit resistance to them [45, 46]. As research progresses, drugs related to anti-angiogenic therapy have emerged, including sorafenib, sunitinib, axitinib, and anlotinib, which target multi-targeted tyrosine kinases [47]. The effectiveness of these anti-angiogenic therapies has been widely demonstrated [48]. Researchers have found that anti-angiogenic therapy improves the tumor vasculature while also improving the state of the immune microenvironment, adjusting the balance between “cold” and “hot” tumor tissues [49]. For example, it has been shown that neutralizing antibodies against VEGFA can effectively enhance the function of CD8^+^ T cells [50] and inhibit the expression of Fas ligand (FasL) on endothelial cells, promoting the infiltration of effector T cells [51]. In addition, the application of specific antibodies blocking angiopoietin 2 and VEGFA for treatment in mouse models of colorectal cancer and melanoma induced vascular normalization and promoted pericyte coverage while effectively promoting T-cell infiltration and aggregation around blood vessels [52]. Not coincidentally, the treatment of mouse colorectal cancer with antibodies against A2V and CD40 promoted an increase in the number of effector T cells within the tumor tissue [53].

Our research group has demonstrated the potential of the small molecule tyrosine kinase inhibitor anlotinib to improve the balance of the immune microenvironment within the tumor by inhibiting PD-L1 expression on VECs [54]. Notably, anti-angiogenic therapy promotes T-cell infiltration in tumors. However, the increased production of IFN-γ by effector T cells mediates immune escape by tumor cells [52]. The mechanism of IFN-γ-mediated immune escape of tumor cells and the potential benefits of combined ICI therapy are discussed in detail by Locquenghien et al. [55]. This phenomenon serves as the basis for combining anti-angiogenic therapy with ICI therapy for achieving improved cancer treatment outcomes.

In a mouse model of lung cancer, the administration of low doses of apatinib, a VEGFR2 tyrosine kinase inhibitor (TKI), in combination with anti-PD-1 mAbs was shown to exert a positive impact on inhibiting tumor progression [56]. The combined therapy was found to alleviate hypoxia, promote the infiltration of CD8^+^ T cells, and reduced infiltration of TAMs, and effectively prolong survival in mice [56].

Another study investigated the potential of erdafitinib (a fibroblast growth factor receptor [FGFR] inhibitor) in combination with anti-PD-1 mAbs in the treatment of lung cancer [57]. The results showed that combination therapy promoted CD8^+^ T cell infiltration and reduced Treg cell infiltration, leading to an enhancement of anti-tumor immunity through the clonal expansion of T cells [57]. Additionally, in the treatment of neuroblastoma, anlotinib was found to improve the efficacy of anti-PD-1 mAbs by promoting vascular normalization via CD4^+^ T cells and by improving the immunosuppressive microenvironment within the tumor [58]. Together, these findings regarding the combination of multiple anti-angiogenic therapies with ICI therapy illustrate the important role of vascular normalization in the process of countering cancer immunoediting.

#### Targeting tumor-associated fibroblasts

CAFs, as important members of the TME, have an important regulatory role in tumorigenesis. They can promote the invasion and proliferation of tumor cells by remodeling the extracellular matrix and releasing a variety of cytokines that interact with other stromal cells and tumor cells [59, 60]. In addition, CAFs express a variety of immunosuppressive ligands such as PD-L1, which are involved in immune regulation within tumor tissues and are also closely associated with the efficacy of immunotherapy and patient prognosis [61, 62]. Given the important role of CAFs in the TME, precision targeted therapy is essential.

For example, fibrogenic activating protein (FAP), a membrane protease highly expressed on the surface of CAFs, is closely associated with fibroblast proliferation, differentiation, and recruitment, while its high expression is suggestive of a poor clinical prognosis [63, 64]. Wang et al. previously showed the potential of utilizing chimeric antigen receptor (CAR)-T cells with FAP to effectively inhibit the growth of tumor cells in a transplanted tumor mouse model, without significant side effects [65]. Additionally, inhibiting the activation signals of CAFs, such as Janus kinase (JAK)-signal transducer and activator of transcription 3 (STAT3) signaling, can comprise an effective therapeutic strategy that may suppress the proliferation of tumor cells and prevent the conversion of normal fibroblasts to CAFs [66]. Another potential approach is the blockade of cytokines that contribute to the formation of CAFs using neutralizing antibodies, such as blocking the immunosuppressive cytokine, TGF-β (can promote the activation and formation of CAFs while maintaining the functional phenotype of CAFs [67]). These findings emphasize the growing interest in CAFs as a potential therapeutic target for remodeling the TIME. This also reflects the research value and significance of CAFs as a potential therapeutic target in clinical practice.

#### Targeting pericytes

Pericytes are structural cells that are widely distributed in the microvascular wall and are also involved in the development, maturation, and remodeling of blood vessels [68, 69]. Tumor-associated pericytes, one of the important components of the TME, are closely related to tumor cell growth, proliferation, and drug resistance [70, 71]. Valdor et al. found that in malignant glioblastoma, tumor-associated pericytes could release cytokines such as IL-10 and TGF-β to influence the antigen presentation ability of antigen presenting cells (APCs), and they could also express PD-L1 to suppress T-cell responses [72]. Although tumor-associated pericytes have been shown to play an immunosuppressive role in the TME, there remains a limited number of therapeutic strategies aimed at targeting pericytes. Zhang et al. showed that targeting C–C motif chemokine ligand 5 (CCL5)-C–C motif chemokine receptor (CCR) 5 effectively inhibited the deoxyribonucleic acid (DNA) damage repair response in glioblastoma cells mediated by pericytes, thereby enhancing the therapeutic efficacy of temozolomide [73]. Furthermore, inhibitors of bone marrow tyrosine kinase on chromosome X (BMX) can effectively target tumor-associated pericytes, cross the blood–brain barrier, and enhance the penetration and effectiveness of chemotherapeutic agents [74]. The targeting of platelet-derived growth factor receptor β (PDGFRβ)-positive pericytes by using TKIs has demonstrated the clearance of pericytes and inhibition of tumor growth in a mouse model [75]. Thus, targeting pericytes is also a reasonable therapeutic modality to remodel TIME.

#### Targeting mesenchymal stem cells

Mesenchymal stem cells (MSCs), a multifunctional class of non-hematopoietic stem cells with self-renewal capacity, can be recruited by tumor cells to tumor sites where they are transformed into tumor-associated MSCs, thereby acquiring a phenotype that promotes tumor cell proliferation and metastasis [76]. MSCs can enable tumor cells to acquire proliferative and anti-aging features by increasing the number of tumor stem cells, increasing bone morphogenetic protein (BMP) production, and activating the P53/P21 pathway [77, 78]. Additionally, in terms of immunosuppression, tumor-associated MSCs can encourage tumor cells to escape from immune cells by releasing IL-10 to downregulate MHC-I expression on tumor cells [79]. On the other hand, tumor-associated MSCs, like other stromal cells, can also promote tumor progression by expressing PD-L1 [80]. Similar to CAFs, MSCs lack obvious markers, and the anti-tumor and pro-tumor effects of MSCs in tumors remain controversial, making targeting MSCs a challenging therapeutic option. Current strategies for targeting MSCs are still focused on targeting tumor growth-promoting and immune-suppressing factors released by MSCs and on inhibiting the recruitment of MSCs by tumor cells, for example, targeting MSCs to produce the C-X-C motif chemokine ligand 12 (CXCL12), inhibiting metastasis of tumor cells, and enhancing the efficacy of ICI therapy [81, 82]. In addition, targeting indoleamine 2,3-dioxygenase (IDO) released from tumor-associated MSCs can also effectively restore the infiltration of CD8^+^ T cells and B cells and enhance the efficacy of ICI therapy [83]. Taken together, the selection of suitable targets will effectively promote the remodeling of the TIME.

### Normalization strategies of immunity

In the TME, there are many immunosuppressive factors that cause tumor cells to evade recognition and attack by the immune system, and among these immunosuppressive factors, immunosuppressive cells and exhausted effector cells are particularly important. By targeting different inhibitory factors with corresponding precision therapy as shown in Fig. 3, the process of cancer immunoediting can be countered.

#### Targeting inhibitory immune receptors

In 2018, Chen's team put forward the concept of immune normalization, emphasizing the importance of identifying defects and dysfunction in the immune response during tumor progression and developing strategies to specifically correct these defects to restore natural anti-tumor immune capacity [84]. These immune normalization strategies typically induce fluctuations in the normal range of immune responses but do not cause permanent damage to normal organs or tissues. Currently, anti-PD1 therapy is superior to immune normalization strategies. Neutralizing antibodies to PD-1 can restore the normal immune response by binding to PD-1 on the surface of immune cells, thus preventing PD-1 from binding to PD-L1 and PD-L2 on tumor or stromal cells [85].

Multiple studies have demonstrated the efficacy and safety of anti-PD-1 therapy as a strategy for immune normalization [86, 87]. However, advanced tumors are associated with a high heterogeneity and aggregation of immunosuppressive cell populations, resulting in the resistance of some tumor tissues to ICI therapy (i.e., pancreatic and prostate cancers). This low efficacy is also associated with a lack of sufficient infiltrating T cells in the tumor tissue [28] or with the vast majority of effector T cells in the terminal exhaustion period rather than the precursor exhaustion period [88].

Therefore, several novel immune checkpoint inhibitors, including LAG3, immunoreceptor tyrosine-based inhibitory motif domains (TIGIT), Tim-3, and VSTA, are in the process of development as a means of overcoming the limitations and inefficiencies of anti-PD-1 therapy.

LAG-3, an immunosuppressive receptor expressed on activated NK cells and T cells, showed significant suppression of T cell function co-expressed with PD-1 in breast cancer [89]. Conversely, LAG-3 deficient CD8^+^ T cells have been shown to exhibit a stronger effector phenotype and a less exhausted phenotype [90]. Targeting the elevated expression of LAG-3 on CD8^+^ and CD4^+^ T cells in the blood and bone marrow of patients with multiple myeloma will help enhance the proliferative and anti-tumor capacity of T cells [91]. In addition, combining anti-LAG-3 mAbs with anti-PD-1 mAbs has been shown to enhance IFN-γ production and the cytotoxic capacity of T cells, leading to robust control of tumor growth [92]. Similarly, administration of PD-1/LAG-3 bispecific antibody also showed a marked increase in the infiltration of CD3^+^, CD8^+^, and CD45^+^ T cells in the TME as well as a significant reduction in tumor progression [93].

TIGIT is also an immunosuppressive receptor found to be expressed on activated T cells and NK cells and is involved in the functional regulation of NK and T cells [94]. Blockade of TIGIT has been shown to reverse the exhaustion of NK cells and enhance the function of CD8^+^ T cells [95, 96]. Concurrent blockade of both TIGIT and PD-1 helps restore CD266 signaling, thereby improving the function of CD8^+^ T cells [97]. Similarly, Tim-3, another immunosuppressive receptor, has been identified as a hallmark of T-cell exhaustion and high levels of Tim-3 expression are commonly used as a predictor of T-cell dysfunction [98].

Our study revealed a negative correlation between Tim-3 expression and the effector function of cytokine-induced killer (CIK) cells [99]. Interestingly, co-expression of Tim-3 with PD-1 usually typically predicts T-cell exhaustion and loss of stemness [100], and combined blockade of Tim-3 and PD-1 has been shown to restore effector T-cell function, produce stronger tumor regression and an enhanced anti-tumor immune response compared to single agent therapies [101]. Furthermore, the emerging immune checkpoint VISTA has been demonstrated to exert non-redundant immunomodulatory functions when combined with anti-PD-L1 mAbs in preclinical studies of CT26 colon cancer and B16 mouse models [102]. This combination therapy was found to effectively increase cytokine production by tumor-specific CD8^+^ T cells, significantly impede tumor growth, and promote long-term survival in mice [102].

Taken together, the combination of immune checkpoint receptor blockers will effectively promote the infiltration and response of effector T cells; however, the side effects of multi-drug combination therapy are greater compared to those of monotherapy. Therefore, the combination regimen needs to be applied more carefully in clinical practice.

#### Targeting immunosuppressive cells

In the tumor immunosuppressive microenvironment, a large number of immunosuppressive cells are present, such as TAMs, MDSCs, Treg cells, and tumor-associated neutrophils (TANs). Both Treg and Breg cells play a crucial role in shaping the tumor immunosuppressive environment, and both types of cells can inhibit the function of effector cells such as CD8^+^ T cells, Th1 cells, and NK cells through expressing PD-L1 and the release of cytokines such as IL-10, adenosine, IL-35, and TGF-β [103–106]. TGF-β released from Breg cells can also promote the proliferation of Treg cells and the expression of FOXP3, accelerating the formation of an immunosuppressive environment [104].

In terms of Treg cells, several therapeutic regimens have been developed to target these cells and enhance anti-tumor effects. For example, anti-CD25 neutralizing antibodies effectively removed CD25^+^ Treg cells from tumor-bearing mice, thereby increasing the number of tumor-infiltrating CD8^+^ T cells [107, 108]. Additionally, infiltrating effector Treg cells within tumor tissue highly express CTLA-4 compared to naïve Treg cells. and the selective clearance of CTLA-4^+^ Treg cells has been shown to result in complete tumor regression in a mouse model [109, 110]. Similarly, targeting specific markers such as glucocorticoid-induced tumor necrosis factor receptor-related protein (GITR), CCR4, and CCR8 has also been shown to selectively eliminate Treg infiltration in tumors or inhibit the migration of Treg cells to the TME, thereby damaging the immune control of Treg cells over effector T cells [111].

In contrast to therapeutic regimens targeting Treg, those targeting Breg cells are still in the early stages of research. The application of anti-CD20 mAbs in order to remove Breg cells has demonstrated good therapeutic efficacy in hematologic tumors, but its efficacy in solid tumors has been limited in solid tumors due to the removal of anti-tumor B cells along with the removal of Breg cells, thus promoting tumor cell proliferation [112]. As an alternative, the application of STAT3 inhibitors to arrest Breg proliferation, reduce the release of IL-10 and IL-35, and relieve the inhibition of effector cells is a promising approach for targeting Breg cells [113, 114].

TAMs and MDSCs play a regulatory role in the tumor immunosuppressive microenvironment similar to that of regulatory lymphocytes. TAMs at the tumor site, stimulated by different cytokines, differentiate into M1-type macrophages (pro-inflammatory phenotype) with anti-tumor effects or M2-type macrophages (anti-inflammatory phenotype) that inhibit anti-tumor effects and release IL-4, IL-10, and IL-13 [115]. Currently, by blocking CD47-signal regulatory protein α (SIRPα) signaling, restoring phagocytosis of tumor cells by macrophages, and promoting the response of effector CD8^+^ T cells, tumor cell proliferation and metastasis are effectively inhibited [116]. Alternatively targeting macrophage growth signals, such as anti-colony stimulating factor 1 receptor (CSF-1R) mAbs, can also effectively improve the size of mouse mammary tumor virus (MMTV)-transgenic polyoma middle T oncoprotein (PyMT) spontaneous breast tumors and prolong the survival of mice by eliminating tumor-resident macrophages or M2- type TAMs [117].

Unlike TAMs, MDSCs, as myeloid-derived suppressor cells, do not exhibit anti-tumor properties and instead exert immunosuppressive effects within tumor tissues. To address this, a therapeutic strategy has been proposed that targets the recruitment of MDSCs from the blood by blocking the corresponding chemokine receptors, such as CCR5 or CXCR2. This approach has been shown to relieve T-cell suppression and inhibit tumor growth and metastasis in melanoma and breast cancer models [118, 119]. Furthermore, the application of conventional chemotherapeutic agents, such as pemetrexed, cisplatin, and paclitaxel, has demonstrated the clearance of MDSCs from tumor sites [120–122]. Similar, blocking the CD40/CD40 ligand (CD40L) signaling pathway on MDSCs would help to inhibit the accumulation of Treg cells and promote the response of effector T cells [123].

In addition to the immunosuppressive cells mentioned above, TANs and mast cells are also important members of the tumor immunosuppressive environment. In the TME, mast cells can accelerate the formation of an immunosuppressive environment by releasing histamine (H1), TGFβ, and IL-10 and inducing angiogenesis [124]. TKIs as the mainstay of mast cell function inhibition have demonstrated their advantages in the treatment of tumors. For example, the inhibition of tyrosine kinase receptor signaling by CD117 will effectively inhibit mast cell recruitment; the inhibition of breakpoint cluster region (BCR)/ABL and protein kinase C (PKC) signaling has also demonstrated the clearance of mast cells and tumor suppression [125–127]. Additionally, the application of H1 receptor antagonists effectively inhibits mast cell infiltration, HIF-1α expression, and tumor growth within melanoma [128].

There are relatively few studies on TANs as an emerging target for improving immunosuppression. In tumor tissues, the number of TANs is significantly increased; they also differentiate into N1 (anti-tumor phenotype) and N2 (pro-tumor phenotype) in response to cytokine and tumor antigen stimulation, which has similarities to TAMs [129]. Similar to mast cells and MDSCs, TANs are mostly recruited from the blood, so blocking the CXCR4-CXCL12 pathway will help inhibit the recruitment of TANs to tumor tissue [130]. Alternatively, tyrosine kinase inhibitors and TGF-β blockers can be used to inhibit the immunosuppressive function of TANs, achieving enhanced immune cell function and inhibiting tumor growth [131, 132].

Taken together, most investigators are still designing strategies to target these immunosuppressive cells around three main aspects: clearance, inhibition of recruitment, and blockade of immunosuppressive factors. However, it is worth noting that most types of immunosuppressive cells share many properties and many targeted drugs can inhibit multiple immunosuppressive cell types. This provides superior anti-tumor efficacy but also poses potential risks for serious side effects, highlighting the need for caution in clinical application.

#### Targeting exhausted immune cells

T cell exhaustion is currently of interest for many researchers. In a TME driven by exhausted immune cells, effector T cells gradually lose their function and eventually become exhausted cells due to the long-term stimulation of chronic inflammation and tumor antigens. Furthermore, such exhausted cells, especially terminally exhausted T cells, are not sensitive to ICI therapy [39]. Progenitor exhausted T cells are a population of T cell factor 1 (TCF-1)-expressing T cells with the ability to self-renew and maintain a long-term response [133]. These progenitor exhausted T cells with high PD-1 expression and low Tim-3 expression are characterized by a rapid proliferative response to exert anti-tumor effects after the application of anti-PD-1 mAbs; however, these cells gradually lose TCF-1 expression and stemness under the continuous stimulation of tumor antigens, thus entering the terminal exhaustion phase [134, 135].

Recent studies have indicated that a complex interplay between epigenetic modifications, metabolic alterations, and perturbed signaling pathways plays a critical role in T cell exhaustion. In the TME, T cell exhaustion is frequently induced by the presence of immune-suppressive cells such as TAMs, MDSCs, and Tregs. These cells exert their suppressive effects by secreting immunosuppressive cytokines, including IDO, TGF-β, and adenosine [136–138].

Furthermore, there is evidence that the synergistic actions of IL-10 and IL-35, which are released by Treg cells, may promote T cell exhaustion in a mouse model of melanoma [139]. Notably, in a chronic lymphocytic leukemia model, a deficiency in IL-10 receptor signaling was found to lead to a reduction in the number of TCF-1^+^CD8^+^ T cells, while an accumulation of PD-1^hi^ led to the exhaustion of T cells, thereby exacerbating tumor progression [140]. This observation highlights the involvement of IL-10/IL-10R signaling in the maintenance of effector T cell stemness.

Interleukin-2 (IL-2), a cytokine pivotal for the proliferation and survival of CD8^+^ T cells, is also critical for memory formation as well as for the maintenance of effector functions of CD8^+^ T cells [141]. Interestingly, persistently elevated levels of IL-2 were found to trigger nuclear translocation of aryl hydrocarbon receptor (AhR) in previous research through sustained activation of the STAT5 pathway, ultimately resulting in CD8^+^ T cell exhaustion [142].

Similarly, previous research has demonstrated that necrotic tumor cells can elevate potassium ion (K +) levels in the tumor interstitial fluid, thereby impeding the mTOR-AKT-mediated effector program in CD8^+^ T cells and leading to their functional exhaustion [143]. Moreover, as research in this domain has progressed, it was discovered that elevated potassium as levels in the tumor interstitial fluid perform a dual role by preserving T cell stemness while restricting T cell effector programs. This is achieved by limiting nutrient uptake by T cells, triggering autophagy, and reducing histone acetylation at exhaustion loci [144].

In addition, lipid metabolism is also implicated in the regulation of T cell exhaustion and the maintenance of T cell stemness. Wu et al. reviewed the effects of lipid metabolism on CD8^+^ T cell function and discussed the paradoxical phenomenon that CD8^+^ T cells require lipids for oxidative phosphorylation for energy, yet excessive fatty acids (FAs) or lipid uptake induces the exhaustion of CD8^+^ tumor-infiltrating lymphocytes (TILs) [145]. It has been shown that TILs with high PD-1 expression in non-small cell lung cancer patients exhibit stronger lipid uptake and higher lipid content compared to TILs with low PD-1 expression [146].

Whereas accelerated fatty acid catabolism may enhance effector functions in CD8^+^ T cells; similarly, inhibition of cholesterol esterification has been shown to improve effector functions and proliferation of CD8^+^ T cells through increased T cell receptor clustering and the formation of immune synapses [147, 148]. However, sustained accumulation of cholesterol in T cells can also induce endoplasmic reticulum (ER) stress, leading to T cell exhaustion [149]. These contrasting results suggest that there is no clear boundary between the maintenance of stemness and the functional exhaustion of effector T cells, and that different exposure durations to the same factor can result in varying outcomes. Therefore, maintaining the stemness of effector T cells or limiting T cells from entering the terminal exhaustion phase has become a central focus of research attention.

In colorectal cancer (CRC), imbalances in urea cycling result in increased levels of extracellular ammonia, leading to oxidative stress in effector T cells and subsequent exhaustion. However, reducing tumor-associated ammonia through targeted interventions reactivates T cells and enhances the effectiveness of anti-PD-L1 therapy [150]. Furthermore, targeting protein tyrosine phosphatase non-receptor type 2 (PTPN2) enhanced the effector function and proliferative capacity of Tim-3^+^CD8^+^ exhausted T cells and improved the efficacy of anti-PD-1 mAbs in treating B16 tumors [151].

Gut microbiome perturbations have been shown to contribute to tumorigenesis by disrupting CD8^+^ T cell homeostasis. This can lead to over-activation of CD8^+^ T cells and eventual exhaustion [152]. However, gut microbial metabolites supplements such as butyrate can enhance effector CD8^+^ T cell responses, via modulation of the ID2-dependent IL-12 signaling pathway, thereby enhancing the effectiveness of anti-PD-L1 therapy [153].

Targeting epigenetics modifications is emerging as a key strategy for preserving T cell stemness. The histone demethylase, lysine-specific demethylase 1 (LSD1), performs an epigenetic program in progenitor exhausted T cells, antagonizes TCF-1-mediated stemness maintenance, and promotes the differentiation of progenitor exhausted T cells toward the terminal phase [154]. Liu et al. found that the maintenance of stemness in progenitor exhausted T cells would be effectively maintained by the genetic perturbation or inhibition of LSD1, enhancing the beneficial effects of anti-PD-1 therapy [154]. In addition, targeting the bromodomain and outer end (BET) protein and DNA methyltransferase 3 alpha (DNMT3A), both of which can affect the program of epigeneticization of T cells, restored the proliferative capacity of exhausted T cells, maintained the anti-tumor capacity of T cells under prolonged antigenic stimulation, and enhanced the killing function of CAR-T cells against tumor cells [155, 156].

Despite limited studies with a focus on targeting exhausted T cells or restoring effector T cell stemness function, it is undeniable that the existing findings are highly encouraging and suggest that this therapeutic approach holds significant potential to achieve immune normalization.

As an alternative to directly restoring the function of exhausted T cells, the choice of adoptive cellular therapy (ACT) is also a reasonable therapeutic strategy in response to the dysfunction of effector T cells in the TME and the insufficient number of infiltrating cells. This treatment strategy is particularly suitable for cancer patients with suppressed autoimmune function after receiving multiple lines of radiotherapy or multilineage therapy. ACT refers to a treatment modality in which autologous or allogeneic immune cells are processed in vitro and then infused back into the patient to inhibit tumor growth, and it is also an emerging treatment strategy in oncology that is unaffected by the immunosuppressive environment in the body and maintains efficient and specific cell-killing properties.

#### Adoptive cellular therapy

Since the first report of ACT in rodents as early as 60 years ago, ACT has developed over the years, including the earlier lymphokine-activated killer (LAK), CIK, NK, and TIL therapies as well as the latest CAR-T therapies [157, 158]. CAR-T therapy is a modification of T cells using genetic engineering techniques to add chimeric antigen receptors to T cells to allow T cells to specifically target tumor cells without the restrictions of the traditional T-cell receptor (TCR)-major compatible complex [159]. Fifth-generation universal CAR-T cells using peripheral blood mononuclear cell (PBMC)-derived T cells from healthy volunteers can obtain a large number of CAR-T cells in a short period of time while being less susceptible to the condition of the patient's immune system and the effects of chemotherapy drugs than autologous CAR-T cells, effectively restoring the normal immune system. In the clinical setting, CAR-T therapy has demonstrated great advantages in the treatment of hematological diseases.

For example, in diffuse large B-cell tumors, three clinical trials demonstrated the efficacy of CAR-T therapy, resulting in CR in 40% of 93 patients [160], 58% of 101 patients, and 53% of 256 patients, respectively [161, 162]. Additionally, in the treatment of multiple myeloma, the efficacy of CAR-T therapy is impressive, with an early clinical trial showing CR in 45% of 33 patients [163]. A meta-analysis also counted 44.8% of 640 patients with multiple myeloma treated with CAR-T who experienced CR [164]. The efficacy of CAR-T therapy in solid tumors was not significant effective compared to that in hematologic neoplasms.

Another ACT is used in the treatment of solid tumors, namely adoptive TIL therapy. As a heterogeneous cell population, TILs, unlike CAR-T cells that target one antigen, can recognize multiple antigens while being less prone to serious immune adverse reactions [165]. The earliest clinical trial of adoptive TIL therapy was in 1988 for the treatment of patients with melanoma [166]. In this study, 9 of 15 patients with metastatic melanoma treated with TIL in combination with IL-2 experienced objective remission and did not experience significant toxicities. The results were groundbreaking and surprising, and they marked the official entry of TIL as a secondary therapy in the field of oncology treatment. Since then, adoptive TIL therapy has been widely used in metastatic melanoma and has improved the objective response rate in unresectable patients from 31 to 72% with improved technology [167]. Adoptive TIL therapy has demonstrated its therapeutic advantages in different cancer types. In the treatment of cervical cancer (9 out of 12 patients achieved CR, NCT04443296) [168] and lung adenocarcinoma (11 out of 16 patients had initial tumor regression one month after TIL injection) [169], adoptive TIL therapy continued to have a good efficacy. In addition, two patients treated with adoptive TIL therapy for breast cancer [170]and colorectal cancer [171] demonstrated good response. Taken together, ACT serves as an important therapeutic tool in immune normalization strategies in response to exhausted cell-driven tumors, and it is currently the most widely studied and applied area.

### Normalization strategies of tumor cells

During the process of cancer immunoediting, tumor cells undergo metabolic and epigenetic reprogramming in response to environmental and physicochemical properties, mutated genes, and other factors, thereby acquiring certain properties that lead to resistance to immunotherapy. Schoenfeld et al. categorized these acquired drug resistance aspects as (1) loss of antigen presentation, (2) loss of IFN-γ signaling, (3) loss of neoantigens, (4) tumor cell-mediated immunosuppression and immune escape, and (5) other immunosuppressive ligands [172]. The vast majority of these aspects are closely related to the mutated oncogene in the tumor cells. Therefore, the choice of a rational treatment induces the transformation of tumor cells into inert tumor cells or normal cells, that is, the normalization of tumor cells. This normalization treatment strategy applies to both oncogene-driven tumors and stromal cell-driven tumor suppressor microenvironments.

#### Induced expression of antigens on tumor cells

In tumor tissues, activated effector CD8^+^ T cells kill tumor cells mainly by recognizing MHC-I molecules on tumor cells. However, the decreased expression of MHCI-like molecules has been observed in the vast majority of human tumor tissues [173, 174]. This loss of MHC molecule expression was found to be closely associated with β2-microglobulin gene mutations, MSI, and defective peptide formation and transport [175, 176]. This decreased expression or loss of MHC-I molecules helps tumor cells to evade recognition by immune cells. In melanoma, reduced MHC-I expression is usually closely associated with disease progression and advanced stages of the disease [177]. In addition to applying anti-CTLA mAbs for metastatic melanoma, MHC-I expression is a crucial factor affecting the efficacy of treatment [178]. Since the majority of tumor cells have low levels of MHC-I expression, inducing elevated MHC expression on the surface of tumor cells helps counter the process of cancer immunoediting and promotes the normalization of immunity.

Chemotherapy, as a classical therapeutic regimen, inherently possesses the property of inhibiting tumor cell value addition and growth, which helps to counter the proceeding of cancer immunoediting. Studies have demonstrated that certain chemotherapeutic agents such as cyclophosphamide, gemcitabine, oxaliplatin, and paclitaxel can enhance the immunogenicity of tumor cells by inducing immunogenic cell death, upregulating the expression of MHC-I on tumor cells and thus enhancing the recognition of tumor cells by immune cells [179–181]. Additionally, vincristine, paclitaxel, and cisplatin can promote the expression of MHC-I on tumor cells by stimulating the production of IFN-β by tumor cells [182].

Radiotherapy, another classical treatment option, is often used in combination with chemotherapy to induce the expression of antigens on tumor cells to enhance the anti-tumor therapeutic effect. Radiotherapy can induce immunogenic death of tumor cells, thus activating both innate and adaptive immunity [183]. In several preclinical studies, radiotherapy has been observed to promote elevated MHC-I expression in tumor cells, enhance T-cell recognition, and effectively kill melanoma as well as lung and breast cancer cells [184–186]. In addition, radiotherapy can lead to the release of DNA from damaged tumor cells, which activates the cyclic guanosine monophosphate (GMP)-adenosine monophosphate (AMP) synthase (cGAS)-stimulator of interferon genes (STING) pathway and thus mediates the release of type 1 IFN, while radiotherapy also activates DCs and stimulates effector T cells to respond to tumor cells [187, 188]. Although radiotherapy and chemotherapy are the main treatment modalities with the ability to counter the proceeding of cancer immunoediting, chemoradiotherapy can also cause corresponding damage to normal immune cells, so the combination of chemoradiotherapy with immunotherapy in clinical practice still requires careful consideration [189].

With advances in research focusing on the epigenetics of tumor cells, Dai et al. have comprehensively reviewed the current effects of epigenetic modifications on the immunogenicity of tumor cells [190]. For example, it has been found that histone deacetylation (HDAC) inhibitors and DNA methyltransferase (DNMT) inhibitors can induce immunogenic cell death and promote tumor cell antigen presentation, MHC-I expression, and tumor-specific antigen production [191, 192]. In addition, targeting EZH2 has been found to promote antigen presentation in tumor cells and reverse resistance to anti-PD-1 therapy by reducing histone H3K27me3 modification on the β-2-microglobulin (B2M) promoter [193]. STING, as an important regulator in tumor immunity, is one of the most hotly studied genes, and the cGAS-STING pathway constituted with GAS is an important DNA sensing mechanism in innate immunity and viral defense. In melanoma cells, deficiency in STING signaling leads to reduced immunogenicity of tumor cells and mediates immune escape of tumor cells, while epigenetic reprogramming of the tumor cell-intrinsic STING function re-induces upregulation of MHC-I expression on melanoma cells and enhances the immunogenicity of tumor cells [194, 195].

In addition to targeting epigenetic drugs, a SMAC mimetic (an IAP inhibitor) was shown to induce elevated expression of MHC-I through the TRAF3/NF-κB pathway, thereby enhancing the sensitivity of tumor cells to T cell-driven cytotoxicity [196]. PCSK9 (a key protein that regulates cholesterol metabolism) was found to promote the re-localization and degradation of MHC-I in lysosomes to disrupt MHC-I recycling at the cell surface, while inhibition of PCSK9 induced a significant increase in MHC-I expression on tumor cells and enhanced the efficacy of anti-PD1 therapy [197].

#### Targeting the inhibitory ligands

PD-L1 (also known as B7-H1 or CD274), one of the ligands of PD-1, binds to PD-1 on effector T cells for immunosuppressive functions and is widely expressed in a variety of tumor cells; the high expression of PD-L1 also predicts poor prognosis [198–200]. Another ligand of PD-1, PD-L2, is mainly expressed on APCs, and it is also expressed in several cancers, including non-small cell lung cancer [201], B-cell lymphoma [202], colorectal cancer [203], and melanoma [204].

Galactose lectin-3 (galectin-3) and liver sinusoidal endothelial cell lectin (LSECtin), ligands for LAG3, are also important immunosuppressive checkpoints expressed on the surface of a variety of tumor cells and are involved in the regulation of CD8^+^ T-cell function [205, 206]. In addition to galectin-3 and LSECtin, fibrinogen-like protein 1 (FGL1) is another important ligand for LAG-3 as a soluble protein that is released by the liver under normal conditions, and FGL1 is highly expressed in a few tumor tissues [207]. Blocking LAG3 has also been shown to help enhance the killing ability of effector T cells [207, 208].

TIGIT is a receptor in the Ig superfamily that plays an important role in adaptive and innate immunity [95, 209]. Its ligands, CD155 and CD112, were found to be expressed on the surface of tumor cells [210, 211]. CD155 expression on the surface of pancreatic duct adenocarcinoma cells is involved in the exhaustion of CD8^+^ T cells, while TIGIT neutralizing antibodies in combination with neutralizing antibodies to PD-1 will contribute to reinvigorating the tumor immune response and effectively inhibit the progression of pancreatic cancer [212].

Carcinoembryonic antigen-related cell adhesion molecule 1 (CEACAM-1), an important ligand for Tim-3, is highly expressed in a variety of advanced tumor tissues. CEACAM-1 induces the downregulation of NKG2D expression in tumor cells, while silencing it in mouse tissues will induce increased ligand expression of NK cells in tumor tissues [213, 214]. Furthermore, CEACAM-1 interacts with Tim-3 through its N-terminal structural domain to form heterodimers in cis and trans to promote Tim-3 maturation and expression on the surface of effector T cells, thereby inducing the generation of immune tolerance [215]. In addition to CEACAM-1, three other ligands are expressed on tumor cells, galectin-9, high mobility group box 1 (HMGB1), and phosphatidylserine (PtdSer). Galectin-9 is highly expressed in hepatocellular carcinoma cell lines and, as one of the ligands of Tim-3, can bind to Tim-3 on Treg cells, thus promoting Treg cells activation and participating in the immune escape of tumor cells [216, 217]. In the TME, PtdSer is exposed to the surface of tumor cells or tumor-derived vesicles and can bind to PtdSer receptors on T cells and macrophages, thereby participating in the immunosuppression of the TME [218].

HMGB1 is more extensively studied than PtdSer and galectin-9. As a highly conserved nuclear protein present in all cell types, HMGB1 is released extracellularly under stress or after cell death, thus activating the innate immune system to participate in the body's inflammatory response [219]. HMGB1 interacts with Tim-3-expressing DCs and inhibits toll-like receptors and cytoplasmic sensor-mediated innate immune responses, thereby hindering the effects of DNA vaccines and chemotherapy [220]. In addition to inhibitory ligands, tumor cells also express FasL (an apoptotic ligand) on their surface to deliver death signals to activated Fas-expressing effector T cells. In contrast, tumor cells themselves are resistant to Fas-mediated apoptotic signals, resulting in a reduced ability of T cells to mediate the killing of tumor cells [221].

CD39 and CD73, key enzymes in the conversion of adenosine triphosphate (ATP) to adenosine, are highly expressed in a variety of hematologic and solid tumors and mediate immune escape from tumors by inhibiting the function of tumor-specific T cells, suppressing the function of Th1 and Th17 cells and enhancing the conversion of type 1 macrophages to type 2 macrophages [222–224].

#### Decrease the viability of tumor cells

The most basic need for tumor cells to survive in the TME is ATP. In the TME, tumor cells undergo metabolic reprogramming in response to mutated genes, hypoxia, low pH values, and multiple growth factors, thus contributing to the fact that tumor cells can acquire energy from the TME in multiple ways, such as carbon skeletons and ATP from glycolysis or mitochondrial oxidative metabolism and de novo nucleotide synthesized from tricarboxylic acid (TCA) intermediates. In the process of energy acquisition by tumor cells, several key enzymes are involved in the reaction, such as glutaminase (elevated expression in several tumor tissues), IDO, and AMP-activated protein kinase. These key enzymes and metabolic pathways contribute to the survival and proliferation of tumor cells, and there are relevant drugs being investigated for these enzymes and metabolic pathways [225–227].

In addition to metabolic reprogramming, tumor cells also exhibit epigenetic reprogramming in response to the TME, resulting in unlimited proliferation and metastatic properties, particularly through DNA methylation. Unlike genetic mutations, epigenetic alterations are reversible as the epigenome can be reprogrammed. As a result, targeting epigenetic regulation in tumor cells to inhibit proliferation and induce apoptosis has become a current focal point in cancer therapy. Several epigenetic therapies have been approved by the United States Food and Drug Administration for tumor treatment. Small molecule inhibitors of DNA methyltransferases have been widely used in the treatment of myelodysplastic syndromes and acute myeloid leukemia, and they have demonstrated significant anti-tumor efficacy in clinical practice [228, 229]. HDAC inhibitors have demonstrated great advantages in the treatment of hematologic tumors; in some patients with peripheral T-cell lymphoma, HDAC inhibitors resulted in longer remissions, with durations of up to 48 months [230]. Current research on epigenetic therapies is also growing more advanced, even pursuing epigenetic reprogramming to influence anti-tumor immunity [231, 232].

In addition to targeting epigenetic regulation and tumor metabolism, therapies targeting oncogenes can also effectively inhibit multiple pathways associated with tumor cells, leading to decreased viability or death of tumor cells, such as those targeting *KRAS* [233], *EGFR* [234, 235], and *HER2* [234]; targeting cell cycle-related genes, such as *CDKs*, *PLK*, *WEE1*, *Aurora A*, and *CHK1*/*2* [236], can also effectively prevent mitosis in tumor cells and reduce their proliferative activity. Furthermore, targeting apoptosis-related proteins, such as B-cell lymphoma 2 (BCL2) [237], can promote apoptosis of tumor cells. Inhibition of multiple pathways can contribute to the decrease in viability of tumor cells themselves and degenerate the process of cancer immunoediting, although it should be noted that these drugs will have notable off-target effects while fighting the tumors. Therefore, the rational use of drugs in the process of countering cancer immunoediting to achieve the maximum anti-tumor efficacy and minimum toxic side effects is also a direction that needs to be further investigated by researchers.

### Combination of normalization strategies

We have discussed three normalization treatment strategies based on cancer immunoediting, namely normalization of tumor cells, normalization of immunity, and normalization of stromal cells. The three normalization strategies can be used either in conjunction with each other or sequentially to maximize the efficacy of the inhibition of tumor progression and to prolong patient survival. For example, the number of infiltrating effector T cells in the microenvironment is increased after anti-angiogenic therapy, while the expression of immunosuppressive ligands on tumor cells and stromal cells increases in the presence of IFN-γ. The above microenvironmental changes provide the prerequisites for the application of ICI therapy. The feasibility of anti-angiogenic therapy combined with ICI therapy has been clinically demonstrated.

The IMpower150 trial was a phase III clinical study demonstrating the benefit of ICI therapy in combination with anti-angiogenic therapy in the first-line treatment of metastatic non-squamous non-small cell lung cancer, both in terms of progression-free survival (PFS) and overall survival (OS) [238]. In the final analysis of the IMpower150 study, a sustained improvement in median OS was demonstrated in the atezolizumab-bevacizumab-carboplatin-paclitaxel group compared to the bevacizumab-carboplatin-paclitaxel group (19. 5 months vs. 14.7 months) [239]. In addition, anlotinib, a small molecule tyrosine kinase inhibitor, has demonstrated good efficacy in combination with ICI therapy in multiple tumor types; for example, a multicenter phase II clinical trial of sintilimab plus anlotinib in PD-L1-positive recurrent and metastatic advanced cervical cancer enrolled 42 patients and found an ORR of 54.8% and a disease control rate of 94.9%. This trial demonstrated the tolerability and efficacy of anlotinib in combination with ICI therapy for cervical cancer [240]. Sintilimab plus anlotinib also demonstrated good treatment efficacy and tolerability in both advanced hepatocellular carcinoma (NCT04052152) and advanced non-small cell lung cancer (NCT03628521) [241, 242].

However, after the application of ICI therapy, some tumor cells showed a loss of response to IFN-γ and a loss of MHC-I expression, thus evading recognition and killing by cytotoxic CD8^+^ T cells [243]. In this case, applying the strategy of tumor cell normalization can effectively restore MHC-I expression on tumor cells and thus enhance the efficacy of ICI therapy. For example, epigenetic drugs, HDAC inhibitors, have demonstrated promising results in promoting immune editing of tumor neoantigens in vivo, as well as enhancing anti-tumor-specific immune responses [244]. In a clinical trial, entinostat (an inhibitor of HDAC) was utilized in the treatment of advanced non-small cell lung cancer that had disease progressed on prior anti-PD-1/PD-L1 therapy, and showed that the entinostat combined with pembrolizumab (an anti-PD-1 mAbs) group demonstrated a robust anti-tumor response, with 9% of of patients had an objective response [245].

**Fig. 4:**
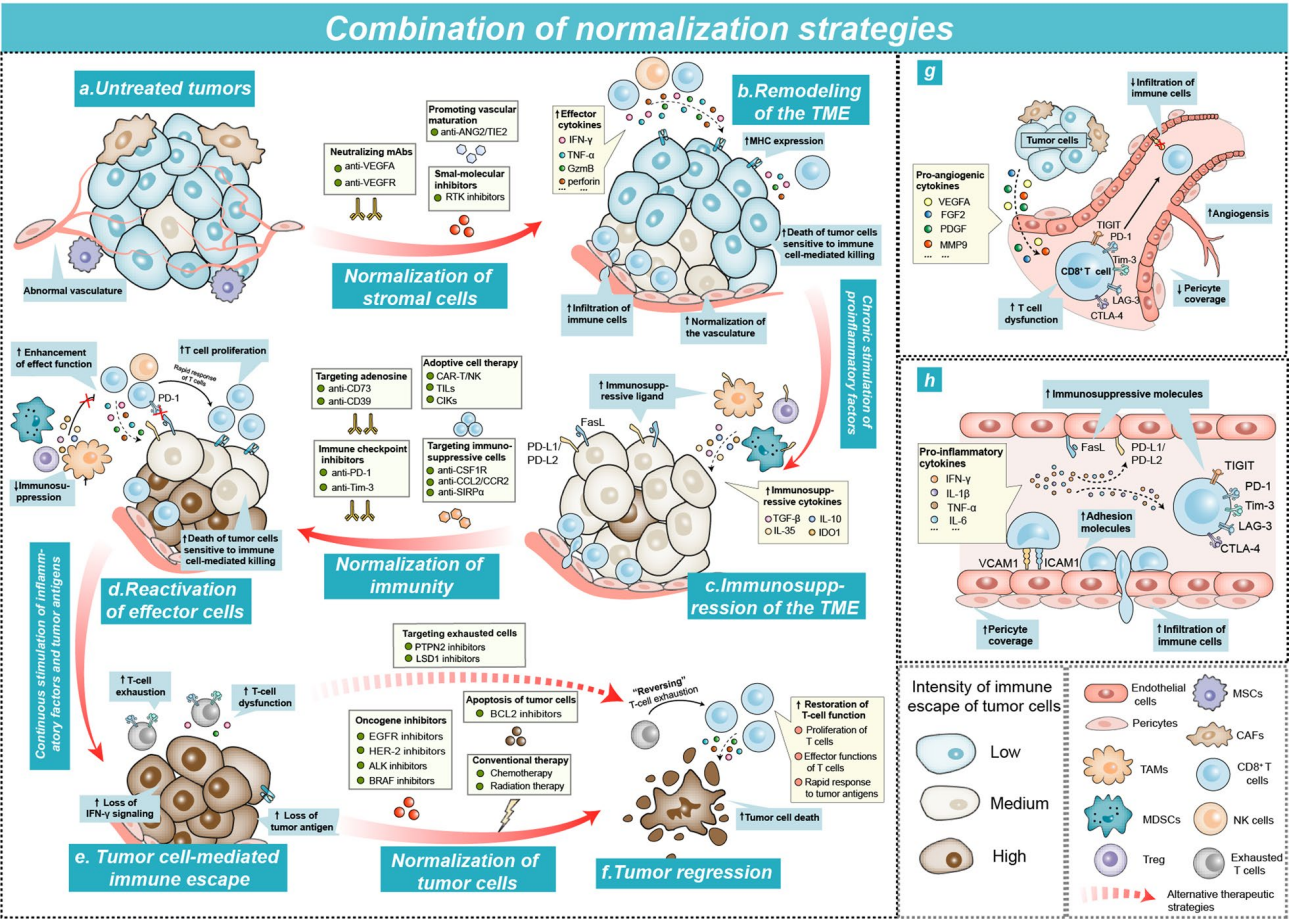
Model of microscopic changes in the TME after normalization therapy. **a**–**f** illustrate a series of changes in the TME resulting from the combined application strategies of normalization therapy. **a** represents the untreated TME; **b** represents the TME that has been remodeled after receiving normalization therapy of stromal cells; **c** represents the TME that becomes immunosuppressive due to the stimulation of continuous pro-inflammatory factors; **d** represents the TME that has undergone normalization therapy of immunity; **e** represents the TME formed by tumor cell-mediated immune escape in the presence of continuous inflammatory factors; **f** represents the tumor regression after normalization therapy of tumor cells; **g** depicts the dynamic changes of immune cells within the vasculature of the untreated TME; **h** depicts the dynamic changes of immune cells within the vasculature after receiving normalization therapy of stromal cells. *TAMs* Tumor-associated macrophages, *CAFs* Tumor-associated fibroblasts, *MDSCs* Myeloid-derived suppressor cells, *MSCs* Mesenchymal stem cells, *RTKs* Receptor tyrosine kinase inhibitors, *VCAM1* Vascular cell adhesion molecule-1. *ICAM1* Intercellular cell adhesion molecule-1, *GzmB* Granzyme B, *IDO* Indoleamine 2,3 -dioxygenase

The above examples demonstrate the promise of the combination of normalization strategies. Based on this, we show the changes in the immune microenvironment under three normalization strategies in Fig. 4 to facilitate readers’ understanding of the advantages of a combination of normalization strategies (BOX 1).

BOX 1 T cells in the proceeding of normalization therapy. During the process of normalization therapy, T cells undergo dynamic changes. Upon receiving stromal cell normalization therapy, such as vascular normalization, T cells are more likely to adhere to the surface of VECs and infiltrate the tumor tissue, leading to a heightened local inflammatory response. This, in turn, promotes high expression of PD-L1 by the tumor cells or stromal cells, suppressing the T-cell response. At this point, most of the T cells infiltrating the tumor site are active and can release pro-inflammatory factors and kill tumor cells. However, these pro-inflammatory factors also result in PD-L1 expression on tumor cells, allowing them to evade killing by immune cells. Further immune normalization strategies can enhance T-cell killing of tumor cells, such as blocking the PD-1/PD-L1 pathway, which promotes the proliferation of T cells with stemness and enhance the killing function of T cells. Over time, continuous antigen stimulation or exposure to pro-inflammatory factors can lead to decreased TCF-1 expression, disappearance of T-cell stemness, and reduced cytokine release capacity. At this stage, the majority of effector cells at the tumor site enter into terminal exhaustion and lose their ability to recognize and kill tumor cells. The strategy of normalizing tumor cells given at this stage can remodel the epigenetics and metabolism of tumor cells, reducing their viability and alleviating T-cell exhaustion. This, in turn, continues to inhibit tumor cell proliferation and may even result in tumor regression.

### Characteristics and counter-immunoediting strategies in the equilibrium phase

#### Adaptive and innate immune systems act synergistically

When the process of cancer immunoediting is in the equilibrium phase, the interaction between tumor cells and the immune system is more insidious. There is a dynamic balance between unsuppressed tumor cells and an immune system that still has immune function, but this phase of equilibrium is relative rather than absolute [1]. According to the previous opinion in the field, the adaptive immune system was involved in the maintenance of this homeostatic state. However, recent experiments suggest that the innate immune system may also be involved, since skin carcinogenesis in the Rag-/- mouse model without adaptive immunity still undergoes cancer immunoediting [246]. In the adaptive immune system, CD8^+^ T cells have long been considered important for the control of tumor growth as a typical effector cell population [247, 248]. It has been shown that gliomas inoculated in mice deprived of CD8^+^ T cells are more likely to become tumors and that gene fusion is more likely to occur in mice burdened with gliomas [249]. Park et al. found that tissue-resident memory CD8^+^ T cells (TRM cells) could help maintain a long-term equilibrium between melanoma cells and the immune system; furthermore, this equilibrium was more easily damaged, and melanoma cells grew more readily in mice that lost TRM cells [250]. These findings demonstrate the synergistic role of the adaptive and innate immune systems in monitoring and inhibiting tumor growth during the equilibrium phase of cancer immunoediting.

#### Long-lasting and undetectable

Most of the tumors in the equilibrium phase do not show obvious tumor features and may even maintain a state of dynamic equilibrium for a long time. Koebel et al. showed that tumor cells in mice treated with the chemical carcinogen methylcholanthrene could remain latent in the body for more than 100 days until immunosuppressive agents were applied and the tumor tissue was detected [251]. In addition to animal experiments, there are also similar cases in the clinic where two patients who received kidney transplants were diagnosed with secondary metastatic melanoma after transplantation. A review of the donor's medical history revealed that the donor had a history of melanoma and had been completely cured for more than 10 years [252]. Furthermore, the time interval between the transformation of Barrett's esophagus into adenocarcinoma of the esophagus is also part of the equilibrium phase, which can last up to 30 years [253]. This phenomenon illustrates the long duration, latency, and unobservable features of the equilibrium phase. Therefore, when the process of cancer immunoediting is in the equilibrium phase, the clinical challenges are confirming whether the patient is in the equilibrium phase and selecting interventions to administer during cancer immunoediting.

#### Diagnosis and treatment of tumors in the equilibrium phase

Because of the lack of characteristic features of tumors in the equilibrium phase, they are not easily detectable in the clinical setting, and therefore there is no clear academic definition of a tumor in the equilibrium phase. We have summarized several tumors that may be in the equilibrium phase below. For example, the majority of melanocytes in the body are benign and do not have the ability to proliferate or divide. Some benign melanocytes can become cancerous in response to ultraviolet light or other external stimuli and transform into melanoma cells, thus regaining the ability to proliferate. The transformation process of normal melanocytes into melanoma refers to the equilibrium phase. During this phase, the malignant melanoma cells interact actively with the immune system; for example, the spontaneous regression of melanoma (20% to 30%) has long been reported, mainly because of strong association between the high heterogeneity of melanoma and the immune response, making it more susceptible to regulation by its own tumor-infiltrating CD4^+^ T and CD8^+^ T cells [254–256]. In addition, researchers found that the spontaneous regression of tumors was closely linked to infection, inflammation, and surgical factors [257]. This also reflects the importance of cancer immunoediting for the interaction between the immune system and tumor cells during the formation and progression of melanoma, as well as where tumors in the equilibrium phase can move to the elimination phase with the intervention of immunotherapy.

Another example is ground-glass nodules or solid nodules of the lung. Since the vast majority of these solid and ground-glass nodules are benign lesions occurring due to inflammation, they are often overlooked, while some of the slow-growing or stable non-resolving ground-glass nodules are considered to be adenocarcinoma in situ or early-stage lung cancer; 29% to 34% of these persistent ground-glass nodules are diagnosed as malignant [258]. However, although ground-glass nodules are histomorphologically malignant, they are often behaviorally inert, and the prognosis of their development into lung adenocarcinoma is usually better. Therefore, clinical practice is mainly based on long-term follow-up observation to avoid excessive medical treatment and waste [259, 260]. Xiao et al. used single-cell sequencing and found that subsolid node lung cancer had a higher infiltration of CD8^+^ T cells than solid node lung cancer, while both had a lower infiltration of NK cells, a feature of subsolid nodules that is consistent with the predominant effect of the adaptive immune system and the impaired innate immune system during the equilibrium phase [261]. During the transition from the equilibrium phase to the escape phase, Wu et al. found that the number of infiltrating CD8^+^ T cells in the synchronized ground-glass nodules of lung cancer patients was significantly lower than that of infiltrating CD8^+^ T cells in tumor tissues and that the infiltration of TAMs in the ground-glass nodules was significantly increased [262]. This point demonstrates the poor immune infiltration in ground-glass nodules and suggests that tumor cells induce the increased infiltration of TAMs in the induction of malignancy. This process shows a possible pattern of transition of tumor cells from the equilibrium phase to the escape phase.

In addition, some investigators have identified T-cell recognition of tumor cell neoantigens as a key to maintaining the equilibrium phase of cancer immunoediting [263, 264]. The intensity of immune response activity within the tumor tissues can also be considered as a marker indicating the transition from the equilibrium phase of the tumor to the escape phase, especially in the immune inflammation-rich (hot tumor) immune microenvironment. Desai et al. believe that this equilibrium is closely related to the TIME. In a hot TIME, immune activity within the tumor tissue is more abundant and sustained, demonstrating that the immune system is actively fighting tumor growth; it also demonstrates a tendency for the tumor tissue to convert to the escape phase [265]. In light of these findings, Desai et al. suggest that immunotherapy for such tumors in the equilibrium phase will effectively maintain the patient's equilibrium phase for as long as possible [265]. However, most tumors in the equilibrium phase are currently treated surgically; these include most precancerous lesions that turn into early cancerous diseases, such as subsolid nodules, junctional nevi, colon polyps, and chronic atrophic gastritis, among others [266, 267].

Treatment strategies that rely on surgical resection of visible cancer lesions and adjuvant chemotherapy are limited in their ability to address the complex process of cancer immunoediting. While these approaches may provide initial tumor control, they do not prevent cancer recurrence or halt the underlying cancer progression, leaving patients at continued risk of disease progression. To address these challenges, a novel strategy of neoadjuvant immunotherapy has been introduced clinically, i.e., systemic immunotherapy followed by surgery. Compared to adjuvant therapy after resection, higher levels of tumor antigens are presented to specific T cells in the circulatory system under neoadjuvant immunotherapy, which can better prevent or counter the process of cancer immunoediting [268]. In 2018, Frode et al. described the results of a clinical trial of neoadjuvant anti-PD-1 mAbs (nivolumab) for resectable early-stage lung cancer (stage I, II, or IIIA) in 21 patients, with 45% of 20 patients observed to have a major pathologic response at the primary tumor site [269]. In addition, the advantages of neoadjuvant immunotherapy in prolonging patient survival have been demonstrated in melanoma [270]and glioblastoma [271]. Therefore, when considering the treatment for tumors in the equilibrium phase, attention should be paid to the status of the systemic immune system and targeted interventions should be performed.

Over the past decade or so, cancer vaccines have not achieved satisfactory results in cancer therapy, and only sipuleucel-T has received FDA approval for its remarkable outcomes in prostate cancer. This limited success has led to the realization that the challenges posed by the highly heterogeneous and immunosuppressive tumor microenvironment in the escape phase severely restricts the effectiveness of vaccines. Saxena et al. summarized the prerequisites for successful cancer vaccine therapy, which include a low tumor burden, a limited immunosuppressive environment, and an active adaptive immune response [272]. This precondition is consistent with the characteristics of the equilibrium phase, where the tumor has a low burden and adaptive immune function is not yet compromised. Therefore, administration of a cancer vaccine during the equilibrium phase may represent a practical and effective therapeutic approach to counter progression in the process of cancer immunoediting.

Okada et al. previously presented a comprehensive review of the current state of research in the field of tumor neoantigens as well as their use in cancer vaccines [273]. In studies conducted on mouse models, neoadjuvant anti-melanoma vaccine treatment has been shown to be more effective in preventing tumor recurrence compared to adjuvant vaccine therapy, demonstrating approximately 100% prevention in tumor recurrence; this difference correlates with an higher frequency of tumor-specific T cells at the time of surgery and an increased number of T cells infiltrating the tumor, lymph nodes, and resected area [274]. In particular, this protective effect was found to be dependent on a CD8^+^ T cell-mediated immune response, and the deletion of CD8^+^ T cells completely eliminated any vaccine-induced anti-tumor immune response in a mouse mesothelioma model [275].

In clinical trials, cancer vaccines have also shown a favorable ability to induce specific immune responses. In a randomized clinical trial of a neoadjuvant vaccine for low-grade gliomas, investigators found that neoadjuvant vaccination induced the expansion of effector CD8^+^ T cells in peripheral blood and prompted vaccine-responsive migration of CD8^+^ T cells into the TME [276]. In a phase II clinical trial (NCT02153918) of the PROSTVA vaccine for prostate cancer (a poxvirus vaccine with a viral vector containing the PSA coding sequence and three co-stimulatory factors), neoadjuvant therapy induced T cell infiltration and systemic immune responses [277]. However, most of the cancer vaccines currently available for neoadjuvant therapy have only demonstrated a favorable safety and pathological remission rate, whereas no promising advantage in survival prognosis has been found.

We note that the mRNA vaccine has gained traction in the fight against COVID-19 and is being explored as a promising option for cancer therapy. Compared to traditional vaccines, mRNA vaccines have the advantage of encoding multiple tumor associated antigens (TAAs) or neoantigens simultaneously, thereby promoting adaptive immune responses, and are more applicable for current personalized treatment approaches [278]. Additionally, mRNA vaccines present the benefits of rapid production, low cost, and high safety, which may help shorten the process of drug development as well as the period of clinical trials. In consideration of the significant benefits of mRNA vaccines, several companies, including CureVac, BioNTech, and Moderna, have accelerated the development of mRNA vaccines, especially personalized vaccines; several products are already in phase I/ II clinical trials, and to date these vaccines show a favorable safety and immune response [279, 280]. In the first reported human application of a personalized mRNA vaccine for melanoma, a neoantigen-specific mRNA vaccine containing 10 patient-specific mutations was injected into the lymph nodes of the enrolled patients and was found to induce an exceptional vaccine-specific immune response in all patients [281]. The cumulative recurrence rate of metastatic events was significantly reduced after vaccination compared to pre-vaccination, and even one patient after relapse achieved CR with anti-PD-1 therapy [281].

Similarly, in another clinical trial (NCT01970358) of six patients receiving a neoantigen-specific vaccine, four patients achieved long-term PFS, while two patients with progressive disease also achieved complete remission after anti-PD-1 therapy [282]. This specific T-cell immune response can persist for several years, amplifying the scope of tumor-specific cytotoxicity in melanoma patients, an thereby contributing to long-term maintenance and countering the progression of the process of cancer immunoediting [283].

Moreover, TAA-based mRNA vaccines have achieved significant breakthroughs in clinical efficacy compared to the largely disappointing clinical outcomes from TAA-based vaccines seen within the past 20 years. In a phase 1 clinical trial, a liposomal RNA vaccine containing four TAA-encoded liposomes for the treatment of patients with unresectable melanoma (NY-ESO-1, MAGE-A3, tyrosinase, and TPTE) induced strong CD4^+^ and CD8^+^ T cells immunity and mediated a durable objective response [279]. Although most mRNA vaccines under study are currently in the early stages of clinical trials, current results have indicated that mRNA vaccines encoding neoantigens and TAAs comprise promising approaches for intervening in the progression of the process of cancer immunoediting during the equilibrium phase.

### Characteristics and counter-immunoediting strategies in the elimination phase

#### Innate immunity performs a major function

When the cancer immunoediting process is in the elimination phase, the innate immune system mainly performs the effector function. Kim et al. discussed the mechanism of tumor cell clearance by the immune system during the elimination phase in their previous review [284]. The process begins with the recognition and killing of tumor cells by innate immune cells, particularly NK cells, and is a rapid and effective process [285]. In contrast, NK cells in mice cleared by anti-asialo-GM 1 mAbs were found to be more likely to induce MCA-sarcoma [286]. Furthermore, the transformation of normal cells into malignant cells or the epithelial mesenchymal transformation of tumor cells can lead to the absence of NK inhibitory signals on the cell surface as well as the upregulation of various ligands of NKG2D and cell adhesion molecule 1; this activates NK cell-mediated specific immune surveillance functions and can effectively inhibit tumor metastasis and malignant transformation of normal cells [287–289].

Similarly, NK cells can recognize tumor cells that lost MHC-I expression using the Ly49 receptor, thus preventing immune escape by tumor cells [290]. Indeed, transgenic mice with Ly49-deficient MMTV-PyTV develop spontaneous mammary tumors faster than mice with proper Ly49 expression, while their tumors are infiltrated with CD69^+^ and have fewer granzyme B^+^ NK cells [291]. In addition, the lack of an immunostimulatory receptor expressed on NK cells, NKG2D, accelerated the progression of Eμ-myc-induced lymphomas [292]. The researchers also found that innate lymphocyte type 3 (ILC3) plays a crucial role in tumor immunosurveillance. In a mouse melanoma model treated with cyclophosphamide and an antibody against tyrosinase-related protein 1 (aTRP1), the combination group exhibited significantly better control of tumor progression compared to the control group, as a result of the accumulation of intra-tumor macrophages promoted by CD90^+^NK1.1^−^ ILC3s [293]. Interesting, nonclassical "patrolling" monocytes were found to have a greater capacity for immune surveillance, with a higher ability to release CCL3/4/5 (which recruits NK cells to clear metastatic tumor cells) compared to classical inflammatory monocytes [294]. Similar evidence describing the role of the innate immune system in the elimination phase has been presented extensively in previous literature [15, 289, 295].

#### Treatment of tumors in the elimination phase

Because of the insidious character of the elimination phase, few studies have shown whether interventions in the immune system during this phase can contribute to a reduction in cancer incidence. O'Donnell et al. believe that the application of vaccines to prevent tumor progression is the holy grail of the elimination phase [14]. The application of vaccines to prevent cervical epithelial neoplasia induced by human papillomavirus infection and hepatitis B virus-induced liver cancer has also been shown to be effective in reducing the incidence of cancers [296, 297]. However, this vaccination mainly promotes the specific killing effect of the adaptive immune system in vivo, thus removing only the virus-infected cells, and it does not significantly enhance innate immunity, which is most important during the elimination phase. Because of this, vaccines are likely not the best method of intervention to use during the elimination phase. A prospective study of 755,459 volunteers in 2020 showed that regular daily exercise helped reduce the risk of seven types of cancers, including colon, kidney, and breast cancers [298]. Similar evidence has been reported in other studies [299, 300]. There are some preliminary studies that suggest an important role for NK cells in the context of exercise and cancer prevention.

Exercise helps increase the number of NK cells in the circulatory system and enhance the ability of the innate immune system to monitor the presence of malignant cells by increasing blood levels of catecholamines [301]. Regular exercise also promotes NK cell toxicity by increasing the release of IL-15 and inducing the expression of NKG2D [302, 303]. In addition, regular exercise can indirectly affect the function of NK cells by influencing physiological changes such as blood perfusion, oxygen consumption, and body temperature [301]. Therefore, regular exercise may be an important intervention that comprehensively upgrades the ability of immune system during the elimination phase, and thus cancer prevention can be achieved. In addition to exercise, proper nutritional intake, mental health, and adequate sleep have been shown to regulate the immune system.

The impact of nutrition and diet on the immune system has been extensively studied and is well established. Deficiencies in vitamins and micronutrients (i.e., zinc, copper, iron) have been shown to impair NK cell activity, macrophage phagocytosis, and the specific killing ability of T cells, and the restoration of optimal levels of these nutrients through dietary supplementation has been demonstrated to effectively enhance immune system performance [304, 305].

In individuals with inadequate nutritional status, supplementation may offer protective benefits against cancer. Conversely, in individuals with adequate nutritional status, the use of supplements may increase the risk of cancer [306]. Excessive nutrient intake contributes to overnutrition, resulting in the conversion and storage of some of the excess nutrients in adipose tissue. Adiposity has a significant impact on the immune system as it can regulate T cell activation and proliferation through the release of leptin. Additionally, leptin can modulate macrophage phagocytosis, cytokine production, and differentiation towards the M1-type macrophages [307, 308].

Han et al. found that white adipose tissue is rich in memory lymphocytes that contribute to immunosurveillance and long-term protective defense [309]. However excessive fat accumulation leads to the development of obesity, which severely impairs the function of the immune system [310]. Obesity induced by a high-fat diet (HFD) induced obesity has been found to alter fatty acid distribution in tumors, reduce the infiltration and function of CD8^+^ T cells, and accelerates tumor growth [311]. Obesity also disrupts the normal cellular metabolism and transportation of NK cells by inducing peroxisome proliferator-activated receptor (PPAR)-driven lipid accumulation, thereby severely impairing their cytotoxicity [312]. Hahn et al. investigated 800 aged mice and found that mice on a long-term restricted diet showed longer survival compared to mice in the casual eating group, highlighting the importance of maintaining a long-term balanced diet and proper nutritional intake for optimal immune function as well as for delaying aging [313].

Similarly, mental and psychological factors are equally important for the immune function of the body. Short-term or acute stress may enhance immune system function; Dopp et al. found that acute stress caused by marital conflict induced an increase in the number of circulating NK cells and CD8^+^ T, as well as improved cytotoxicity of NK cells [314]. Additionally, short-term stress can enhance macrophage and CD8^+^ T cell function and migration capacity, as well as immune memory, through affecting the concentrations of adrenal hormones and various cytokines (e.g., IL-1a, TNF, and IL-6) [315]. And chronic stress, depression, and anxiety will suppress immune function; in these states, the central nervous system induces the production of high levels of plasma corticosteroids, which inhibit the function of macrophages and T cells [316]. Studies have also found that during severe mood swings, such as during the death of a spouse, corticosteroid levels are significantly increased and NK cell activity is significantly suppressed [317]. Therefore, maintaining mental health is an important strategy for preserving the normal function of the immune system.

Circadian disruption was identified as a possible carcinogen as early as 2007 [318]. Namely, disturbed circadian rhythm and sleep disturbance may significantly reduce melatonin levels and is strongly associated with breast carcinogenesis [319]. Cortés-Hernández et al. suggest a noteworthy relationship between circadian rhythms and the modulation of immune evasion by circulating tumor cells (CTCs), namely that CTCs adjust their activity in response to unfavorable portions of the circadian cycle, reducing or limiting their activity, and conversely, by enhancing their activity during favorable periods [320]. Recently, Diamantopoulou et al. confirmed the speculation that CTCs are more prone to metastasis during sleep than during wakefulness, a phenomenon that occurs in association with key circadian hormones such as melatonin, testosterone and glucocorticoids [321]. This finding does not indicate that sleep is an accomplice in promoting tumor progression, but instead indicates only that CTCs are more likely to enter the bloodstream at a particular phase of the circadian rhythm. Furthermore, sleep disturbances and disruptions in circadian rhythms may also seriously affect immune function; for instance, 10 days after vaccination against influenza, study participants who experienced multiple days of sleep restriction (4 h of sleep) had influenza virus-specific antibody titers that were less than half the level of those of participants allowed to maintain a normal sleep duration [322]. Similarly, sleep deprivation for only 24 h produced a similar decrease in antibody titers during hepatitis A vaccination; in contrast, adequate sleep was shown to promote an increase in the number of antigen-specific CD4^+^ T cells and the formation of immune memory [323, 324]. Therefore, maintaining adequate sleep and a normal circadian rhythm remains an important strategy for enhancing the function of the immune system during the elimination phase.

**Fig. 5:**
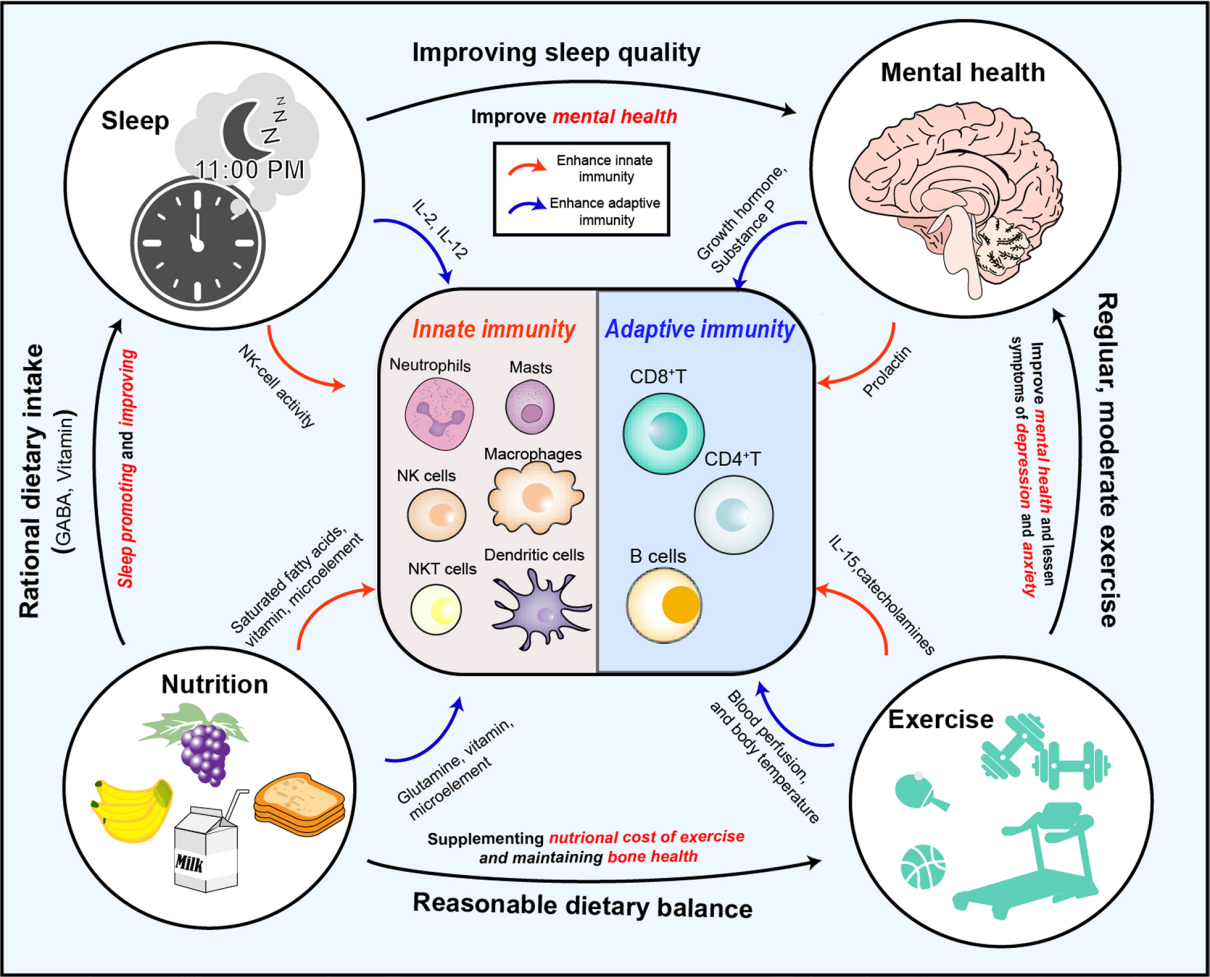
The impact of a healthy lifestyle on the immune system during elimination phase. Mental health, exercise, nutrition, and sleep synergistically enhance the function of the immune system during the elimination phase. *GABA* Gamma-aminobutyric acid

In addition, Exercise, nutrition, mental health, and sleep are not only involved in the regulation of the immune system but are also closely related. Regular exercise and adequate sleep are known to improve mental health and decrease stress, anxiety, and depression [325, 326]. An adequate nutritional intake helps sustain regular exercise and improve sleep quality [327, 328]. Taken together, interventions in the elimination phase should target these four aspects to develop a lifestyle of regular exercise, healthy psychology, adequate sleep, and rational nutritional intake. This intervention pattern will strongly enhance innate and adaptive immunity and arrest cancer immunoediting in the elimination phase (Fig. 5). However, it will require ongoing research efforts to translate these findings into practical clinical treatments.

## Conclusion

**Fig. 6:**
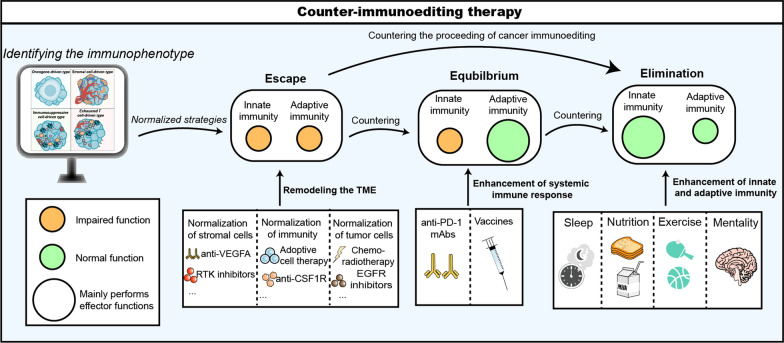
The process of counter-immunoediting therapy. Patients are first phenotyped from the initial diagnosis to understand their immune status, followed by the selection of appropriate normalized treatment strategies according to different phenotypes; When the escape phase retrogrades to the equilibrium or elimination phase, the corresponding treatment strategy is selected for further intervention to help the immune system effectively target and kill tumor cells. This approach has the potential to enable patients to achieve a CR or maintain a long-term cure by taking into account the patient's unique immune phenotypes and selecting the most appropriate treatment strategy. *RTK* Receptor tyrosine kinase inhibitors, *TME* Tumor microenvironment

The theory of cancer immunoediting explains the paradoxical relationship between tumor cells and the immune system. Based on this paradoxical relationship, we propose a set of personalized and precise immunotherapy methods and protocols, i.e., by identifying the phase of cancer immunoediting and selecting the rational therapeutic strategy for each stage, thus promoting the retrogradation of cancer immunoediting and ultimately the complete regression of the tumor (Fig. 6). Furthermore, we also performed a more detailed typing of the immune microenvironment and proposed three normalization therapeutic strategies for these immunotypes. These three normalization therapeutic strategies can be applied not only individually, but also in a certain order to maximize the survival benefit for cancer patients.

In summary, according to the immune microenvironment typing and treatment concept proposed in this review, unlike the previous single-target, single-drug, multi-line treatment model, counter-immunoediting therapy will provide patients with normalized therapeutic strategies in a targeted manner in the clinical setting, considering the patient's overall immune microenvironment. Meanwhile, we have summarized the main therapeutic strategies and approaches involved in counter-immunoediting therapy in Table 1.

**Table 1:** Impact of multiple therapeutic strategies and approaches for counter-immunoediting therapy on innate and adaptive immunity

Phase	Treatment strategies	Approaches	Target/interventions	Total effect	Impact: adaptive immunity	References	Impact: innate immunity	References
Escape phase	Normalization strategies for stromal cells	Targeting VECs and neovascularization (Suitable for stromal cell-driven type and oncogene-driven type)	Target: VEGF/VEGFR, PDCF/PDGFR, EGF/EGFR, etc	Promotes normalization of vasculature and facilitates infiltration of effector cells	Enhances the function of CD8^+^ T cells; promotes the infiltration of CTL and CD4^+^ T cells; reduces the infiltration of Treg cells	[50, 58, 329]	Promotes the infiltration of NK cells; prevents the dysfunction of NK cells; reduces the recruitment of TAMs; promotes the functional maturation of DCs; reduces the number of tumor-associated mast cells	[8, 56, 330–332]
		Targeting CAFs (Suitable for stromal cell-driven type and oncogene-driven type)	Target: Wnt2, FAP, mesothelin, TGF-β, HIF2, NetG1, etc	Enhances the function of effector cells and remodels the TME	Restores anti-tumor T-cell responses; enhances the function of CD8^+^ effector T cells; decreases the number of tumor-infiltrating Tregs; inhibits the formation of Treg cells	[333–335]	Promotes the infiltration and activation of NK cells; reduces the recruitment of M2 macrophages; increases the number of active DCs; inhibits the infiltration of mast cells	[333, 336–338]
		Targeting pericytes (Suitable for stromal cell-driven type and oncogene-driven type)	Target: DLK1, IL-33, IL-10, TGFβ, PD-L1, etc	Enhances the function of effector cells and remodels the TME	Enhances anti-tumour T-cell responses; promotes the activation and recruitment of CD8^+^ T cells; reduces the infiltration of Treg cells	[72, 339]	Reduces the recruitment of TAMs	[340]
		Targeting MSCs (Suitable for stromal cell-driven type and oncogene-driven type)	Target: IDO, IL-6, IL-8, IL-10, galectin-1, PGE2, activin-A, etc	Enhances the function of effector cells and remodels the TME	Promotes the proliferation and infiltration of T cells; promotes the recognition of tumor cells by CTL; promotes the infiltration and differentiation of B cells	[79, 83, 341]	Enhances NK cell-mediated cytotoxicity; inhibition of M2 macrophage polarization; restores the differentiation and function of DCs; promotes the infiltration and activation of mast cells ;	[342–346]
	Normalization strategies of immunity	Targeting inhibitory immune receptors (Suitable for immunosuppressive cell-driven type and stromal cell-driven type)	Target: PD-1, TIGIT, VISTA, LAG3, Tim-3, CTLA-4, etc	Relieves immunosuppression of effector cells and restores the function of immune cells	Promotes the infiltration of effector T cells; maintains and enhances the function of effector T cells; inhibits the infiltration of Treg cells; enhances the function of B cells	[91, 93, 96, 347, 348]	Prevents NK cell exhaustion; enhances the NK cell-mediated cytotoxicity	[95, 349]
		Targeting immunosuppressive cells (Suitable for immunosuppressive cell-driven type and stromal cell-driven type)	Target: TAMs, Treg, TANs, mast cells, Breg, etc	Relieves immunosuppression mediated by immunosuppressive cells; restores and enhances the immune function of effector cells	Promotes the activation, proliferation, infiltration and function of effector T cells; inhibits the function and proliferation of Treg cells; inhibits the function of Breg cells	[104, 108, 109, 113, 123, 132]	Promotes the activation, infiltartion, proliferation and function of NK cells; restores the phagocytic function of macrophages; inhibitis the infiltration of M2-type macrophages; promotes the transformation from M2 type to M1 type; promotes the activation of DCs; inhibits the infiltration of tumor-associated mast cells	[116, 117, 128, 350–354]
		Targeting exhausted immune cells (Suitable for exhausted T cell-driven type and stromal cell-driven type)	Target: PTPN2, LSD1, CISH, TIGIT, tumor-associated ammonia, gut microbiota, etc	Reverses the function of T cell exhaustion	Promotes the proliferation and activation of T cells; reverses the exhaustion of T cells; enhances the effector function and proliferative capacity of Tim-3^+^CD8^+^ exhausted T cells; maintains the effector T cells stemness	[150, 151, 154]	Promotes the activation, infiltartionn and function of NK cells; prevents NK cell exhaustion	[95, 355]
	Normalization strategies of tumor cells	Induced expression of antigens on tumor cells (Suitable for oncogene-driven type)	Interventions: chemotherapy, radiotherapy, DNMTi, EZH2i, HDACi, IAPi, etc	Enhances the recognition of tumor cells by effector cells	Promotes tumor cell recognition and killing by T cells; enhances the function of T cells; promotes the infiltration of effector T cells; reduces the numbers and function of Treg cells	[180, 191–194, 196, 197, 356]	Restores the function of NK cells; promotes the activation, function and maturation of DCs	[180, 188, 356]
		Targeting the inhibitory ligands (Suitable for oncogene-driven type and immunosuppressive cell-driven type)	Target: PD-L1, PD-L2, galectin-3, LSECtin, FGL1, CD155, CD112, CEACAM-1, galectin-9, HMGB1, PtdSer, FasL, CD73, CD39, etc	Relieves tumor cell-mediated immunosuppression and enhances the function of effector cells	Promotes the activation, proliferation infiltration and function of effector T cells; prevents T cell apoptosis; inhibits the activition of Treg cells; promotes the activation of B cells	[198, 205, 207, 212, 215, 216, 357]	Promotes cytotoxic capacity and maturation of NK cells; enhances engulfment of macrophages; promotes the transformation from M2 type to M1 type; promotes the activation of DCs; inhibits the activiation of tumor-associated mast cells	[213, 358–363]
		Decrease the viability of tumor cells (Suitable for oncogene-driven type and exhausted T cell-driven type)	Interventions: Targeting metabolic and epigenetic reprogramming, targeting oncogenes, targeting cell cycle-related genes, targeting apoptosis-related proteins, etc	Reduces the immune escape ability of tumor cells and restores normal immune function	Prevents T cell apoptosis and enhances effector T cell function; enhances the infiltration and reactivation of tumor-specific T cell; reduces the infiltration of CD4^+^ effector regulatory T cells; promotes the infiltration of B cells	[364–367]	Supports the NK cell-mediated cytotoxicity; promotes the infiltartion of NK cells; inhibits the activation of TAMs; inhibits the activation of mast cells	[367–370]
Equilibrium phase		Neoadjuvant therapy	Target: PD-1	Promotes intratumoral and systemic adaptive immune responses	Increases the number of T-cell clones; induces T-cell activiation	[269, 271, 371]	Induces cDC1 activation	[371]
		Cancer vaccine	Target: Neoantigen, TAAs, TSAs, etc Interventions: TLR and STING agonist, CD40 agonist, Oncolytic viru, etc	Promotes systemic adaptive immune responses	Induces strong CD4^+^ and CD8^+^ T cell immune responses; promotes the antigen-specific T-cell responses; promotes the infiltration of T cells; reduces the number of intratumoral Treg cells	[279–281, 372]	Augments the cytotoxic function of NK cells; promotes the transformation from M2 type to M1 type; promotes the activation and maturation of DCs	[372–374]
Elimination phase		Regular exercise	Interventions: Endurance exercise, aerobic exercise, exercise training, etc	Enhances the function and mobilization of NK cells in the bloodstream, maintains their ability to perform immune surveillance	Promotes the mobilization and accumulation of tumor-infiltrating IL15Rα^+^ CD8^+^ T cells; enhances the function of CD8^+^ T cells; increases in absolute numbers of Tregs; increases the recruitment of Treg; promotes the mobilization of immature B cells	[375–379]	Promotes the infiltration and activation of NK cells; reduces the number of TAMs; regulates the polarization of TAMs; preferentially mobilizes DCs; promotes the infiltration and activation of mast cells	[380–384]
		Rational nutritional intake	Interventions: Micronutrient supplements	Enhances adaptive and innate immune system responses	Promotes the maturation and proliferation of T cells; protects T cells from apoptosis; promotes the proliferation and effector fonction of B cells	[305, 385–388]	Enhances NK cell cytotoxic activity and maintains its function; promotes the inflammatory of macrophages; promotes the maturation and differentiation of DCs; restores tumor-associated DC functionality; maintains the stability of mast cells	[305, 389–393]
		Mental health	Interventions: Psychological interventions	Mainly enhances the activity of NK cells and maintains the immune surveillance ability of NK cells	Promotes the proliferation and activition of T cells; promotes the production of antibodies	[394–396]	Maintains or enhances NK cell cytotoxic activity; restores the function of DCs; inhibits the function and infiltration of mast cells	[317, 395, 397–399]
		Adequate sleep	Interventions: Maintains adequate sleep	Enhances the function of adaptive immunity, promotes the formation of immune memory, and maintains the function of the innate immune system.	Promotes an increase in the number of antigen-specific CD4^+^ T cells and the formation of immune memory; promotes the production of antibodies	[323, 324]	Supports the activity of NK cells	[400]

*VECs* Vascular endothelial cells, *CAFs* Tumor-associated fibroblasts, *MSCs* Mesenchymal Stem Cell, *TAMs* Tumor-associated macrophages, *CTL* Cytotoxic T cells, *DCs* Dendritic cells, *TAAs* Tumor associated antigens, *TSAs* Tumor specific antigens, *TME* Tumor microenvironment, *HDACi* HDAC inhibitor, *DNMTi* DNMT inhibitor, *IAPi* IAP inhibitor, *EZH2i* EZH2 inhibitor; *TANs* Tumor-associated neutrophils

We believe that with the further development of artificial intelligence technology and more comprehensive assessment of tumor immune microenvironments from multiple dimensions, the therapeutic strategy of counter-immunoediting therapy will have a wider application prospect, making cancer immunotherapy the most promising method for curing cancer.

## Data Availability

Not applicable.

## Abbreviations

NK: Natural killer cells
TRM: Tissue-resident memory CD8^+^ T cells
HPV: Human papillomavirus
TAMs: Tumor-associated macrophages
MSI: Microsatellite instability
PFS: Progression-free survival
OS: Overall survival
DCs: Dendritic cells
CTL: Cytotoxic T cells
TILs: Tumor-infiltrating lymphocytes
TMB: Tumor mutation burden
ICIs: Immune checkpoint inhibitors
VECs: Vascular endothelial cells
CAFs: Tumor-associated fibroblasts
ECM: Extracellular matrix
FAP: Fibrogenic activating protein
TANs: Tumor-associated neutrophils
ACT: Adoptive cellular therapy
ICD: Immunogenic cell death
MDSCs: Myeloid-derived suppressor cells
DNMT: DNA methyltransferases
AML: Myeloid leukemia
HADC: Histone deacetylation
CR: Complete remission
ORR: Objective response rate
IDO: Indoleamine 2,3 -dioxygenase
OA: Oncolytic adenovirus
VCAM1: Vascular cell adhesion molecule-1
ICAM1: Intercellular cell adhesion molecule-1
TAAs: Tumor associated antigens
A2V: Neutralizing antibodies to VEGFA and ANGPT2
ER: Endoplasmic reticulum
